# A fluoride-responsive genetic circuit enables in vivo biofluorination in engineered *Pseudomonas putida*

**DOI:** 10.1038/s41467-020-18813-x

**Published:** 2020-10-07

**Authors:** Patricia Calero, Daniel C. Volke, Phillip T. Lowe, Charlotte H. Gotfredsen, David O’Hagan, Pablo I. Nikel

**Affiliations:** 1 The Novo Nordisk Foundation Center for Biosustainability, Technical University of Denmark, 2800 Kgs, Lyngby, Denmark; 2 School of Chemistry, University of St. Andrews, KY16 9ST St, Andrews, UK; 3 Department of Chemistry, Technical University of Denmark, 2800 Kgs, Lyngby, Denmark

**Keywords:** Metabolic engineering, Applied microbiology, Synthetic biology

## Abstract

Fluorine is a key element in the synthesis of molecules broadly used in medicine, agriculture and materials. Addition of fluorine to organic structures represents a unique strategy for tuning molecular properties, yet this atom is rarely found in Nature and approaches to integrate fluorometabolites into the biochemistry of living cells are scarce. In this work, synthetic gene circuits for organofluorine biosynthesis are implemented in the platform bacterium *Pseudomonas putida*. By harnessing fluoride-responsive riboswitches and the orthogonal T7 RNA polymerase, biochemical reactions needed for in vivo biofluorination are wired to the presence of fluoride (i.e. circumventing the need of feeding expensive additives). Biosynthesis of fluoronucleotides and fluorosugars in engineered *P*. *putida* is demonstrated with mineral fluoride both as only fluorine source (i.e. substrate of the pathway) and as inducer of the synthetic circuit. This approach expands the chemical landscape of cell factories by providing alternative biosynthetic strategies towards fluorinated building-blocks.

## Abstract (web summary)

Addition of fluorine to organic structures is a unique strategy for tuning molecular properties, but approaches to integrate fluorometabolites into the biochemistry of living cells are scarce. Here, the authors develop a fluoride-responsive genetic circuit to enable in vivo biofluorination in engineered *Pseudomonas putida*.

## Introduction

The chemistry of halogens is essential for key aspects of our current lifestyle. Fluorine (F), in particular, is indispensable for industrial applications in the pharmaceutical, agricultural, and material sectors^1–3^. Almost 25% of all pharmaceutical molecules contain F atoms^4,5^, incorporated chemically during drug development to improve pharmacokinetics thereby increasing bioavailability. The latest figures indicate that the market for organic fluorinated compounds (organofluorines) will continue to expand considerably in the coming years^6^. F is the most electronegative element in the Periodic Table and elemental fluorine (F_2_) is extremely reactive; conversely, this high electronegativity imparts an inert property to aqueous fluoride ion (F^−^)—rendering it difficult to participate in carbon–fluorine bond forming events with the manifold of naturally evolved enzyme mechanisms^7^. As a consequence, naturally occurring organofluorine metabolites are exceedingly rare^8,9^. The development of approaches for the site-selective introduction of F into structurally diverse molecules would circumvent the harsh chemical methods associated with its introduction in organic chemistry, and remains one of the great goals in metabolic engineering^10^. The discovery of the fluorinase enzyme^11,12^ [5′-fluoro-5′-deoxyadenosine (5′-FDA) synthase] in *Streptomyces* and related Gram-positive bacterial species offered a unique opportunity to address this challenge. This is the only enzyme known to incorporate inorganic fluoride (F^−^) into organic compounds by catalyzing the S_N_2 addition of F to the universal C1 donor *S*-adenosyl-L-methionine (SAM), thereby generating 5′-FDA, which then undergoes phosphorolysis by a purine nucleotide phosphorylase (PNP) to generate 5′-fluoro-5′-deoxy-D-ribose 1-phosphate (5′-FDRP). The final fluorometabolites of the route in *Streptomyces* *cattleya* are fluoroacetate or 4-fluorothreonine^13^.

The kinetic properties of the fluorinase enzyme have been characterized in vitro^14^, and most efforts in enzyme engineering thus far aimed at improving catalytic efficiency, increasing activity and widening substrate range^15^. The biotechnological exploitation of this enzyme is hampered mostly because of the toxicity of either F^−^ or some organofluorines in microbial hosts and the very slow kinetics of the fluorinase. The very few examples of in vivo fluorometabolite production include heterologous expression of the fluorinase gene in the marine actinomycete *Salinospora tropica* to obtain the anticancer drug fluorosalinosporamide^16^—although the recombinant strain showed very significant growth inhibition even at very low F^−^ concentrations. A fluorinase gene was recently introduced into *Escherichia coli*, but further strain manipulations and external feeding of SAM were required to promote detectable 5′-FDA synthesis^17^. Other cases of fluorometabolite biosynthesis involved addition of already fluorinated precursors or enzyme cofactors to the culture medium, e.g., as substrates for engineered polyketide synthases^18,19^ or toward the incorporation of F atoms into polyhydroxyalkanoates in engineered *E*. *coli*^20^. Establishing de novo biofluorination in a robust, genetically tractable cell factory for cost-effective fluorometabolite biosynthesis, independent of feeding organofluorine or expensive precursors and inducers, is still a major challenge in metabolic engineering.

In this study, we have engineered the soil bacterium and platform strain *Pseudomonas putida* KT2440 as a cell factory for de novo biofluorination toward biosynthesis of 5′-FDA and 5′-FDRP. *P*. *putida* is a model host for industrial biotechnology that can accommodate reactions involving toxic substrates and products that virtually no other bacterial host can handle^21–24^, and we have turned our attention to this bacterium to engineer biofluorination pathways that involve e.g. toxic F^−^ salts. To this end, we combine variants of the fluorinase and PNP enzymes from different *Streptomyces* species, displaying enhanced activity in Gram-negative bacteria, with a tightly regulated F-responsive riboswitch and the orthogonal T7 RNA polymerase, to create a synthetic circuit in which NaF, the actual substrate for biofluorination, triggers the formation of the products of interest (fluoronucleotides and fluorosugars). In addition, we validate the use of the synthetic circuit as a F^−^ biosensor in vivo, and we demonstrate its application to enable the biosynthesis of 5′-FDA and 5′-FDRP in engineered *P*. *putida*, activated by the addition of a readily-available inorganic salt to the culture medium. De novo fluorometabolite biosynthesis in a surrogate bacterial host in the absence of externally added precursors thus provides access to bio-based production of organofluorines, expanding the catalytic scope of cell factories beyond its natural boundaries.

## Results

### Riboswitch-based, F^−^-responsive expression system for bacteria

**Fig. 1:**
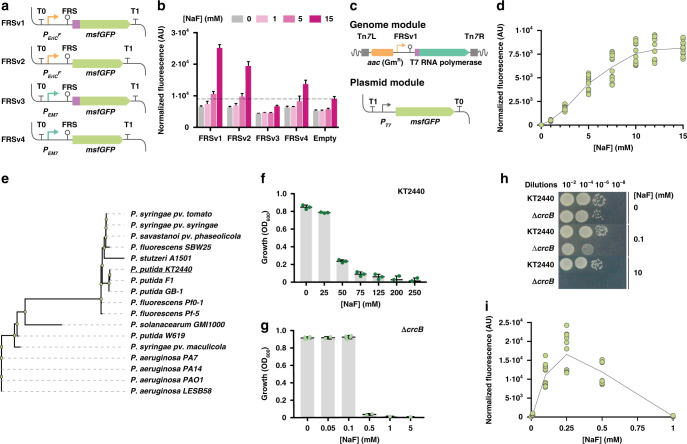
Construction and characterization of a fluoride-responsive gene circuit. **a** Constructs bearing the F^−^ responsive riboswitch (FRS) with different combinations of the native *eriC*^*F*^ promoter (*P*_*eriC*_^*F*^, orange arrows) or a constitutive, synthetic promoter (*P*_*EM7*_, dark green arrows) and the sequence encoding the first eight amino acids of EriC^F^ (purple box) were tested. The *msfGFP* sequence and transcriptional terminators are indicated in light green and T symbols, respectively. **b** Normalized fluorescence of *P. putida* KT2440 transformed with the FRS constructs shown in (**a**) in response to different NaF concentrations. The fluorescence baseline (empty vector) is marked with a dashed line; data are presented as mean values and error bars represent standard deviations of three different biological replicates. (**c**) Synthetic circuit based on the FRS and the T7 RNA polymerase. FRSv1 regulates the expression of the T7 RNA polymerase gene (dark green), encoded in a mini-Tn*7* module (left and right sites of mini-Tn*7* depicted as dark gray boxes), along with a gentamycin resistance cassette (orange). This module is genome-integrated in *P*. *putida*; a plasmid module containing *P*_T7_ → *msfGFP* (pS231T7::msfGFP) was transformed in the same strain. **d** Normalized fluorescence of *P. putida* KT2440::FRS-T7RNAP/pS231T7::msfGFP exposed to increasing NaF concentrations. Data are presented as mean values and error bars correspond to standard deviations from three biological replicates. **e** Phylogenetic tree for CrcB proteins in *Pseudomonas* species. **f** Toxicity of F^−^ in *P. putida* KT2440 grown in M9 minimal medium with different NaF concentrations. Bacterial growth (optical density at 600 nm, OD_600_) is shown after 20 h of incubation. Error bars represent standard deviations of three biological replicates. **g** Same as in (**f**), but with *P. putida* KT2440Δ*crcB*. Error bars represent standard deviations of at least *n* = 2 different biological replicates **h** Tolerance of *P. putida* KT2440 and *P. putida* KT2440Δ*crcB* to NaF exposed by spotting culture dilutions onto LB medium plates containing 0, 0.1, and 10 mM NaF. **i** Same as in (**d**), but with *P. putida* KT2440Δ*crcB*::FRS-T7RNAP. Data are presented as mean values and error bars correspond to standard deviations from three biological replicates. Source data underlying **b**, **d**, **f**, **g**, **i** are provided as a Source Data file.

Efficient bioproduction of chemicals demands significant efforts to reduce costs derived, among other factors, from the additives needed for protein expression (e.g., inducers or antibiotics)^25^. Riboswitches are regulatory elements found in the 5′-UTR of bacterial mRNAs, exerting post-transcriptional control of gene expression upon binding to a specific metabolite^26^. Riboswitches have been adapted as biosensors, affording easy screening of high-producer microbial strains^27^. However, the widespread use of these regulatory devices is limited by their strong contextual effect on gene expression^28,29^. A fluoride ion (F^−^)-responsive riboswitch (FRS) has been described in bacteria and archaea, where it regulates the expression of genes involved in ion detoxification—typically controlling F^−^ transporters^30^. The sequence, structure and mechanism of action of FRS have been described in *Pseudomonas syringae* and *Bacillus subtilis*^31^. We explored if a minimal FRS structure could be adapted as an orthogonal expression system in *P*. *putida*, which would afford the use of basal F^−^ salts as inducers of gene expression. To this end, we adopted the FRS sequence from *P. syringae* (closest relative to strain KT2440, which would support transferability of the cognate genetic parts) as a structural template for a family of DNA constructs. In its natural context, the FRS drives the expression of *eriC*^*F*^ (encoding a putative F^−^ exporter). Four variants of an FRS expression cassette were constructed where *msfGFP*, encoding the monomeric superfolder GFP, is placed under control of a hybrid promoter-FRS element (using either the synthetic P_*EM7*_ promoter or the native P_*eriC*_^*F*^ promoter of *P. syringae*), with or without the leading sequence of the EriC^F^ transporter fused to the msfGFP-coding sequence (Fig. 1a; Supplementary Tables 1,2 and Supplementary Data 1). Expression cassettes were insulated by means of the T0 and T1 transcriptional terminators, and plasmids containing each variant were transformed into *P*. *putida* KT2440. The resulting recombinants were grown in M9 minimal medium containing glucose and NaF at increasing concentrations, and msfGFP fluorescence was detected as readout (Fig. 1b). The synthetic FRS element reacted to the addition of NaF, spanning a range of 1.5- to 3.8-fold induction. Considering this poor dose-response behavior, we constructed a synthetic circuit to connect the FRS-dependent signal in the FRSv1 module (which showed the best performance among all four versions) to the activity of the orthogonal T7 RNA polymerase. To this end, we transferred the FRSv1 module (i.e., the promoter region of *eriC*^*F*^, the riboswitch element and nucleotides encoding the first seven amino acids of EriC^F^) upstream of the gene-encoding T7 RNA polymerase (Fig. 1c). This synthetic circuit would allow for the expression of the orthogonal RNA polymerase, while keeping the FRS genetic context intact (a critical factor for riboswitch performance^29^, which could also account for the poor performance of the other FRS constructs tested). The synthetic FRS-T7RNAP module was integrated as a single copy into a transcriptionally neutral locus in the genome of *P. putida* KT2440 downstream of *glmS* by means of a mini-Tn*7* delivery vector. The resulting strain, *P. putida* KT2440::FRS-T7RNAP, can be used to host constructs where the *P*_*T7*_ promoter controls the expression of virtually any gene(s) of interest. We used plasmid pS231T7::msfGFP, in which the gene encoding msfGFP is cloned under regulation of the *P*_*T7*_ promoter, to examine the performance of this engineered *P. putida* strain (Fig. 1c). Cells harboring this dual system (i.e., chromosomally integrated FRS-T7RNAP and plasmid-borne *P*_*T7*_ → *msfGFP*) were cultured in M9 minimal medium with glucose, and gene expression was induced by addition of NaF at different concentrations. NaF did not affect bacterial growth up to 15 mM, and increasing F^−^ concentrations led to higher msfGFP fluorescence levels following a sigmoidal trend (i.e., according to the classical Hill equation), yielding a discernable msfGFP output above background levels already at 2.5 mM NaF (Fig. 1d). The msfGFP fluorescence was very low in the absence of NaF (even below the levels observed for the single-plasmid system, Fig. 1b); an indication that the dual system is tightly regulated, displaying low levels of basal expression. The highest NaF concentration tested (15 mM) led to a 200-fold increase in the fluorescence output (the induction range for the single-plasmid system was ca. fourfold when tested under similar conditions), and was kept at the base concentration for further biofluorination assays. These results suggest that the transcriptional T7 RNA polymerase-dependent circuit broadens the dynamic range of the FRS-T7RNAP module, also allowing for a tighter regulation of gene expression. However, an intriguing feature of the behavior of both the single and dual system based on FRS is the relatively high NaF concentration needed to trigger activity. Thus, the dynamics of these expression systems was studied in *P*. *putida* as indicated in the next section.

### CrcB controls intracellular levels of F^−^ in *P. putida*

Since we observed relatively low msfGFP fluorescence levels in the experiments above, we wondered whether the intracellular F^−^ concentration in *P*. *putida* could be a limiting factor for the activity of the F^−^-triggered synthetic FRS system. F^−^ toxicity has been studied in other bacterial species, and we set to identify key factors governing F^−^ homeostasis in strain KT2440. In silico inspection of its genome^32^ suggested that the product of *crcB* (*PP_4001*) could act as a F^−^ transporter. The orthologue in *E. coli* K-12 mediates F^−^ removal, keeping low intracellular ion concentrations^33^. Interestingly, *crcB* orthologues are widespread in microorganisms, and present in most of the fully sequenced *Pseudomonas* strains (Fig. 1e), displaying a sequence clustering that separates fluorescent pseudomonads (e.g., *P*. *putida* and *P*. *fluorescens*) from pathogenic *P*. *aeruginosa* and related species. An in-phase, scarless deletion of *crcB* was implemented in *P. putida* KT2440, and the effects of eliminating *crcB* were assessed by evaluating NaF toxicity in both the wild-type strain and the Δ*crcB* mutant. *P. putida* KT2440 thrived in M9 minimal medium in the presence of NaF at 75 mM (although the final cell density was reduced to ca. 10% of that in the control experiment, without NaF; Fig. 1f). Growth of the Δ*crcB* mutant, in contrast, was abruptly suppressed by any NaF concentration above 0.5 mM (Fig. 1g). Moreover, the growth-inhibition effect of increasing NaF concentrations in the wild-type strain followed a decreasing asymptotic trend, whereas a small change in the extracellular concentration of F^−^, from 0.1 to 0.5 mM, completely inhibited the growth of the Δ*crcB* mutant. Solid media assays (i.e. cell suspensions were spotted onto agarized M9 minimal medium containing glucose and different NaF concentrations) confirmed these findings—highlighting that the growth of the Δ*crcB* mutant was almost indistinguishable to the wild-type strain in the absence of NaF (Fig. 1h). These assays were repeated in the presence of NaCl, and no such deleterious effect was detected, indicating that CrcB is a selective F^−^ transporter that plays a crucial role in the survival of *P. putida* KT2440 when exposed to this ion.

### The FRS-T7RNAP/PT7 → msfGFP circuit as F^−^ biosensor

Considering that intracellular F^−^ levels are kept low by the CrcB transporter, we tested the dual FRS-T7RNAP/*P*_*T7*_ → *msfGFP* system as a biosensor of the intracellular F^−^ concentration in *P*. *putida*. To this end, we constructed strain *P. putida* Δ*crcB*::FRS-T7RNAP by delivering the module into the chromosome of the mutant, and we then introduced the plasmid carrying *P*_*T7*_ → *msfGFP* into the resulting strain. When the engineered strain was cultured in M9 minimal medium with glucose, quantifying msfGFP fluorescence as the output, the system responded to very small changes in the external NaF concentration (Fig. 1i). No leaky expression of *msfGFP* could be detected in the absence of NaF, while the addition of 100  μM NaF triggered a response in the biosensor comparable to that in the wild-type strain when exposed to the maximum NaF concentration (Fig. 1d). The output of the system further increased ca. 1.5-fold when the external concentration of NaF reached 250 μM. As expected, raising the level of the salt higher to 1 mM in the culture medium severely affected growth (hence, biosensor performance) as the Δ*crcB* mutant is unable to counterbalance the toxic effects of F^−^. The output of the FRS-T7RNAP/*P*_*T7*_ → *msfGFP* biosensor in the two strains under scrutiny (Fig. 1d and i) suggests that the intracellular concentration of F^−^ in *P*. *putida* KT2440 would be ca. 0.1 mM when NaF is externally added at 15 mM. With this information at hand, we adapted our system to control F^−^, which plays a dual role as both the trigger and as the base substrate for in vivo biofluorination as outlined below.

### Testing in vitro biofluorination in *P. putida*

**Fig. 2:**
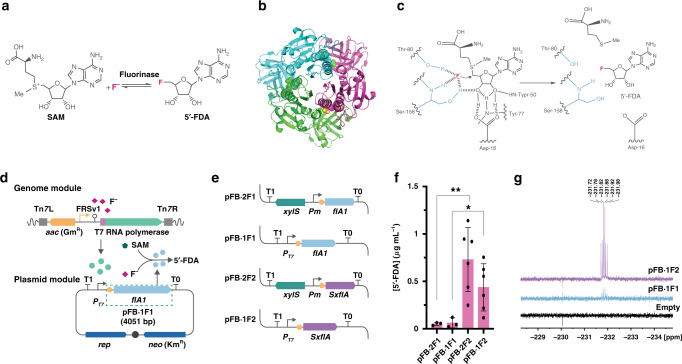
In vitro biofluorination in cell-free extracts of engineered *P*. *putida*. **a** Biofluorination reaction, indicating the transformation of SAM (*S*-adenosyl-L-methionine) and F^−^ into 5′-FDA (5′-fluoro-5′-deoxyadenosine) by the fluorinase enzyme. **b** 3D structure of the trimer form of fluorinase (PDB ID 1RQP); SAM bound to the enzyme is represented as yellow spheres. **c** Mechanism of action of the fluorinase enzyme. Functional groups that interact with F^−^ (magenta) are indicated in blue. **d** Scheme of the synthetic circuit designed for fluoride-triggered biofluorination. The T7 RNA polymerase gene (dark green) is integrated in the genome; upon exposure to F^−^ (pink squares), the *P*_T7_ → *flA1* module (light blue, borne by plasmid pFB·1F1) is expressed and the FlA1 fluorinase (light blue circles) is produced, catalyzing the conversion of SAM (green pentagon) and F^−^ into 5′-FDA. FluoroBrick 1, containing an RBS (yellow circle) and *flA1*, is boxed with dashed green lines. **e** FluoroBricks tested for 5′-FDA biosynthesis. Genes encoding fluorinases from *Streptomyces* sp. MA37 (*flA1*) and *S. xinghaiensis* (*SxflA*) were combined with the XylS/*Pm* expression system or the FRS-T7 RNA polymerase circuit described in (**d**) by modular assembly. **f** 5′-FDA biosynthesis in cell-free extracts of *P*. *putida* KT2440 bearing the FluoroBrick constructs described in (**e**). Data are presented as mean values and error bars correspond to standard deviations of at least three different biological replicates. An unpaired t-test analysis was used to compare 5′-FDA biosynthesis across experiments, with one asterisk indicating a *p* value = 0.0410 and two asterisks identifying a *p* value = 0.0121. **g** ^19^F-NMR spectra of the cell-free extracts. Key chemical shifts corresponding to 5′-FDA are indicated. Source data underlying Fig. 2f are provided as a Source Data file.

The fluorinase of *S*. *cattleya* catalyzes the formation of 5′-FDA from SAM and inorganic F^−^, releasing l-methionine in an S_N_2 substitution process (Fig. 2a). The crystal structure of the enzyme identified a trimer as the catalytically active form (Fig. 2b), which involves three key amino acid residues for catalysis at each of the three active sites^34^ (Asp16, Thr80, and Ser158; Fig. 2c). A survey of the literature identified seven potential fluorinases, with experimental evidence indicating that those of *Streptomyces* sp. MA37^35^ and *Streptomyces* *xinghaiensis*^36^ display the faster kinetic properties in vitro. To test if any of these fluorinases could mediate in vivo biofluorination in *P*. *putida*, we adopted a ‘brick’ approach that enables easy swapping of individual parts (i.e. promoters, RBSs, coding sequences and tags; Supplementary Fig. 1). Such ‘FluoroBrick’ design also allows for composable designs and portability of the modules into other bacteria. The synthetic FRS-T7RNAP/*P*_*T7*_ circuit was included as a functional part of the design (Fig. 2d), and we compared the performance of the XylS/*Pm* system, a well-known expression platform for *P. putida*^37^, against the synthetic circuit for all FluoroBricks encoding fluorinases (Fig. 2e).

Plasmids bearing these constructs were introduced either in *P*. *putida* KT2440 (for XylS/*Pm*-based expression) or KT2440::FRS-T7RNAP (for FRS-T7RNAP/*P*_*T7*_ circuit-based expression), and the strains were cultured in M9 minimal medium with glucose until the mid-exponential growth phase. At this point, the expression of the corresponding FluoroBrick was triggered by the addition of 3-methylbenzoate (XylS/*Pm* system) or NaF (FRS-T7RNAP/*P*_*T7*_ circuit). Cells were harvested at 20 h post induction, cell-free extracts were prepared and immediately tested by incubating them in the presence of SAM and NaF. Considering that 5′-FDA is not commercially available, we optimized a protocol for the chemical synthesis of this fluorometabolite to be used as an analytical standard. The chemical synthesis used 2′,3′-isopropylidene-6-chloropurine riboside as substrate (Supplementary Fig. 2), and 5′-FDA obtained through this method was characterized by LC–MS (Supplementary Table 3) and ^1^H-, ^13^C-, ^15^N- and ^19^F-NMR (Supplementary Table 4 and Supplementary Fig. 3) to confirm its chemical identity and purity. As expected, no 5′-FDA could be detected in cell-free extracts obtained from *P*. *putida* cells transformed with empty vectors. All constructs containing FluoroBricks encoding fluorinase enzymes enabled formation of 5′-FDA in vitro (Fig. 2f and Supplementary Fig. 4a). In particular, heterologous expression of the fluorinase gene from *S*. *xinghaiensis* resulted in the highest 5′-FDA titers (up to ca. 0.4 μg mL^−1^) for all of the configurations tested, with a similar titer attained with both XylS/*Pm*- and FRS-T7RNAP/*P*_*T7*_ circuit-based expression of the fluorinase gene. Furthermore, ^19^F-NMR analysis confirmed the chemical identity of 5′-FDA as the only fluorometabolite present in the samples (Fig. 2g). These results indicate that the FluoroBricks encoding fluorinases result in biofluorination in cell-free extract assays; it then became an objective to assess whether 5′-FDA could be synthesized in vivo.

### Fluoride-triggered in vivo biofluorination in *P. putida*

**Fig. 3:**
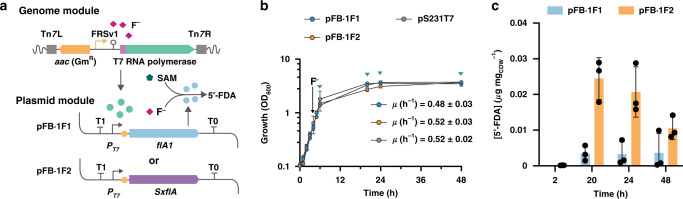
In vivo biofluorination in engineered *P*. *putida*. **a** Scheme of the FRS-T7 RNA polymerase circuit controlling the expression of the two fluorinases *flA1* and *SxflA* in plasmid modules. **b** Growth curves of *P. putida* KT2440::FRS-T7RNAP containing the plasmids described in (**a**), compared to the growth of *P. putida* KT2440::FRS-T7RNAP transformed with an empty vector (pSEVA231). The timing of F^−^ addition to the cultures is indicated with a black arrow. Green arrows indicate the points when cultures were harvested to test 5′-FDA biosynthesis. Mean values and standard deviations of the specific growth rates (μ) after the addition of F^−^ of each construction are shown in the plot. Error bars correspond to standard deviations of three biological replicates. **c** Intracellular 5′-FDA concentration after 2, 20, 24, and 48 h of induction of the synthetic circuit with NaF. Data are presented as mean values and error bars correspond to standard deviations of three different biological replicates. CDW cell dry weight. Source data underlying Fig. 3b, c are provided as a Source Data file.

Growth curves revealed that the presence of plasmid pFB·2F1 (XylS/*Pm* → *flA1*) negatively affected bacterial growth (both in terms of specific growth rate and final cell density) even in the absence of 3-methylbenzoate, the chemical inducer of this expression system. The known leakiness of the XylS/*Pm* system^38^, together with the burden imposed by addition of an aromatic chemical inducer, could hamper the implementation of biofluorination in bacterial cell factories. We reasoned that the synthetic FRS-T7RNAP/*P*_*T7*_ circuit could provide a solution to this challenge since the inducer of the system is the defining substrate (F^−^) for the biofluorination process, thus linking the activities encoded in the FluoroBricks to substrate availability in addition to low leakiness (Fig. 1d). To test this possibility, FluoroBricks encoding fluorinases from *Streptomyces* sp. MA37 and *S*. *xinghaiensis* were cloned under transcriptional control of the *P*_*T7*_ promoter. The resulting plasmids or the empty pSEVA231 vector, used as control, were transformed into *P*. *putida* KT2440::FRS-T7RNAP (Fig. 3a). As hinted above, the expression of these fluorinase genes did not require adding solubility tags to the coding sequences, and growth patterns of the corresponding engineered strains suggested that the constructs impose no metabolic burden on the cells (Fig. 3b), whether in the absence or presence of NaF. In order to explore in vivo biofluorination, NaF was added to all cultures at 15 mM during mid-exponential growth to trigger FluoroBrick expression, also providing the inorganic fluoride needed for the fluorinase reaction. Note that the SAM needed for these transformations is endogenously produced, and no other additive besides NaF was included in these experiments. The intracellular content of 5′-FDA in engineered cells was evaluated at 2, 20, 24, and 48 h by LC–MS analysis (Fig. 3c), and no intracellular 5′-FDA could be detected prior to NaF addition in any of these experiments. Engineered *P*. *putida* containing the synthetic FRS-T7RNAP/*P*_*T7*_ → *flA*^*S*. *xinghaiensis*^ construct displayed the highest fluoronucleotide content, peaking at ca. 0.025 μg mg of CDW^−1^ at 24 h and slightly decreasing at 48 h. In contrast, cells bearing the FRS-T7RNAP/*P*_*T7*_ → *flA1*^*Streptomyces* sp. MA37^ circuit had a 5′-FDA content <0.01 μg mg_CDW_^−1^ throughout the cultivation period. These results confirm the feasibility of using the synthetic FRS-T7RNAP/*P*_*T7*_ circuit to promote biofluorination in vivo triggered by an inorganic inducer in the absence of any expensive additive (e.g., SAM) or chemical inducer of the expression system (i.e., 3-methylbenzoate).

To explore the potential of *P. putida* Δ*crcB* as a background for biofluorination (considering the high intracellular F^−^ concentration in the mutant), we implanted the fluoride-triggered circuit in this strain and tested 5′-FDA biosynthesis as explained above. *P. putida* KT2440Δ*crcB*::FRS-T7RNAP was transformed with the *P*_*T7*_ → *flA*^*S*. *xinghaiensis*^ construct and grown in minimal M9 medium, triggering the expression of the fluorinase gene by addition of NaF at 5 mM. The presence of this salt resulted in an immediate growth arrest both for the engineered strain and the mutant carrying the empty vector (Supplementary Fig. 5a), which meant that NaF could be added to the culture only once sufficient biomass levels to carry out the biofluorination reaction were reached. 5′-FDA was still produced under these conditions, but the fluoronucleotide content reached values ca. 1000 lower than those observed in experiments with the wild-type strain (Supplementary Fig. 5b), ruling out the use of the Δ*crcB* mutant as a host for biofluorination reactions. This occurrence is probably due to the delicate balance that should be met between F^−^ concentrations as an inducer of the FRS-T7RNAP circuit and as a substrate for the fluorinase reaction. The high K_*m*_ for F^−^ by FlA (around 8 mM^34^) indicates that a high concentration of the ion is needed to support biofluorination, but such F^−^ levels seriously compromise cell physiology in the Δ*crcB* mutant. Taking these considerations into account, and having verified 5′-FDA formation by an engineered *P*. *putida*, our focus now extended to the biosynthesis of fluorosugars as indicated below.

### Biosynthesis of fluorosugars in engineered *P. putida*

**Fig. 4:**
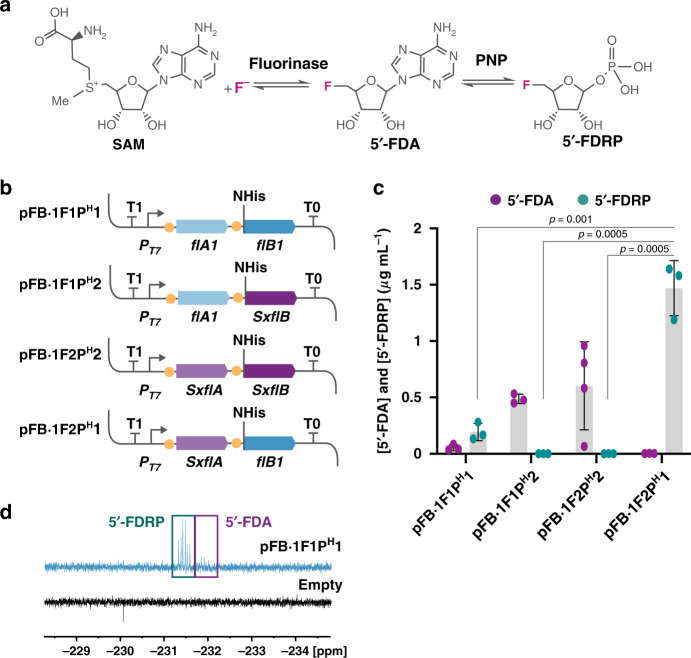
In vitro biosynthesis of fluorosugars in cell-free extracts of engineered *P*. *putida*. **a** Fluorination and phosphorylation steps in the canonical biofluorination pathway of *S. cattleya*. In the second step, 5′-fluoro-5′-deoxyadenosine (5′-FDA) is converted into 5′-fluoro-5′-deoxy-D-ribose 1-phosphate (5′-FDRP) by PNP (purine nucleoside phosphorylase). **b** Plasmid constructs of FluoroBricks encoding fluorinases and PNPs from *Streptomyces* sp. MA37 (*flA1* and *flB1*) or *S. xinghaiensis* (*SxflA* and *SxflB)* under control of the *P*_*T7*_ promoter, which is used in combination with the synthetic FRS-T7 RNA polymerase circuit. Phosphorylases were added with a 6× histidine tag in the *N* terminus as indicated. **c** 5′-FDA and 5′-FDRP biosynthesis in cell-free extracts of *P*. *putida* KT2440::FRS-T7RNAP bearing the FluoroBrick constructs described in (**b**). Data are presented as mean values and error bars correspond to standard deviations of at least three different biological replicates; An unpaired *t*-test statistical comparison of the data identifies differences in 5′-FDRP biosynthesis between samples, *p* values are shown in the figure. **d** ^19^F-NMR spectrum of fluorometabolites in cell-free extracts of engineered *P*. *putida* expressing *flA1* and *flB1* using the synthetic circuit, compared to the spectrum of cells bearing the empty plasmid. Source data underlying **c** are provided as a Source Data file.

The second step in the canonical biofluorination route involves the conversion of 5′-FDA into 5′-FDRP catalyzed by PNP (Fig. 4a). Synthesis of fluorosugars has only been reported in *Streptomyces* and closely related species (i.e., natural producers of fluorometabolites), and we decided to expand the portfolio of fluorinated compounds produced in *P*. *putida* by engineering 5′-FDRP biosynthesis. Several PNP enzymes were assayed to this end, and we found that they are hardly produced in a soluble and active form in *E*. *coli* or *P*. *putida* (Supplementary Fig. 5). To address this issue, the FluoroBrick structure was further expanded to include standard solubility tags that can be incorporated to extend the *C*- or *N*-termini of target proteins (Supplementary Fig. 1). Adding a His tag to the N terminus of the PNP of *Streptomyces* sp. MA37 allowed for the detection of phosphorylase activity, and the constructs were integrated with the fluorinase genes described above to find a combination resulting in the highest conversion of SAM and F^−^ into 5′-FDRP (Fig. 4b). Furthermore, we prepared an analytical 5′-FDRP standard by treating chemically synthesized 5′-FDA with a commercial PNP, and a LC–MS method was developed for detection of fluorosugars using this analytical standard (Supplementary Table 3 and Supplementary Fig. 4b). The constructs containing PNP genes were placed under transcriptional control of the synthetic FRS-T7RNAP/*P*_*T7*_ circuit and transformed into *P*. *putida* KT2440::FRS-T7RNAP. Initial prototyping of this set of FluoroBricks was carried out in cell-free extracts exposed to SAM and F^−^ as explained above, and we found that the combination of the fluorinase from *S*. *xinghaiensis* and the His-tagged PNP from *Streptomyces* sp. MA37 resulted in the highest 5′-FDRP output (Fig. 4c). Interestingly, using *flA* and *flB* genes from the same species hardly resulted in any catalytic activity in vitro (5′-FDRP concentrations <0.02 μg mL^−1^), whereas mixing the *SxflA* and *flB1* modules enabled 5′-FDRP biosynthesis up to ca. 1.5 μg mL^−1^ with no 5′-FDA accumulation (as observed for all of the three other combinations tested). ^19^F-NMR analysis of selected samples confirmed the chemical identity of 5′-FDRP (Fig. 4d) and highlighted the presence of 5′-FDA (i.e., incomplete transformation of SAM and F^−^ into the fluorosugar) when using either a combination of FlA and PNP enzymes of the same *Streptomyces* species or the *flA1* and *SxflB* modules (Fig. 4c). The versatility of the synthetic FRS-T7RNAP/*P*_*T7*_ circuit to trigger biofluorination was further exposed by comparing the output of the same FluoroBricks cloned under control of the XylS/*Pm* expression system. Cell-free extract assays carried out with these constructs indicated poor catalytic performance (Supplementary Fig. 6a), always resulting in mixtures of 5′-FDA and 5′-FDRP at very low concentration (Supplementary Fig. 6b), probably due to the toxicity of 3-methylbenzoate observed previously. With these tools at hand, the next step was to test in vivo biosynthesis of 5′-FDRP using NaF as the only additive supplied to cultures of the engineered strains.

### In vivo biosynthesis of 5′-FDRP in *P. putida*

**Fig. 5:**
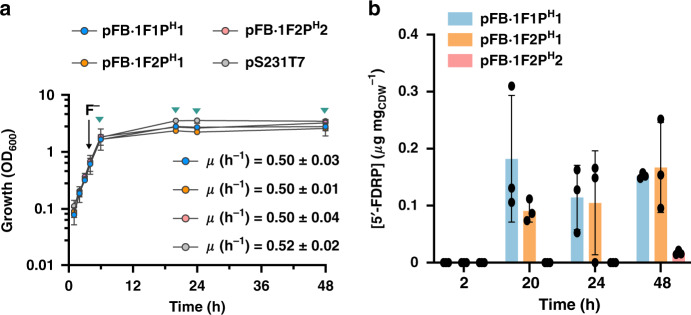
In vivo biosynthesis of fluorosugars in engineered *P*. *putida*. **a** Growth curves of *P. putida* KT2440::FRS-T7RNAP containing the plasmids modules described in Fig. 4b, compared to the growth profile of *P. putida* KT2440::FRS-T7RNAP bearing an empty vector (pSEVA231). F^−^ addition to the cultures is indicated with a black arrow. Green arrows indicate the points when cultures were harvested to test 5′-FDRP biosynthesis. Average and standard deviations of the specific growth rates (μ) after the addition of F^−^ are shown in the plot for each engineered strain. Error bars correspond to standard deviations of three different biological replicates. **b** Extracellular concentration of 5′-FDRP after 2, 20, 24, and 48 h of induction of the synthetic circuit with NaF. Error bars correspond to standard deviations of three different biological replicates. CDW cell dry weight. Data are presented as mean values and error bars correspond to standard deviations from three biological replicates. Source data are provided as a Source Data file.

FluoroBrick constructs tested for fluorosugar biosynthesis were then assayed in vivo by growing *P*. *putida* KT2440::FRS-T7RNAP in minimal M9 medium containing glucose and inducing the expression of FlA and PNP genes with NaF when the cultures reached the mid-exponential phase of growth. Similar to the observations made for in vivo 5′-FDA biosynthesis, the presence of these synthetic circuits did not affect the cell physiology (Fig. 5a). Almost no 5′-FDRP was detected when the intracellular content of fluorosugars was analyzed by LC–MS, and the fluorometabolite was mostly found in culture supernatants. Traces of 5′-FDA (below the limit needed for quantification) were also detected in some supernatants. We reasoned that this phenomenon could be due to either transport via a yet unidentified exporter or to increased membrane permeability due to the presence of F^−^. To shed some light on this behavior, we conducted a propidium iodide assay under biofluorination conditions to stain *P*. *putida* cells with compromised membranes (Supplementary Fig. 7). Interestingly, exposure of all cultures to NaF resulted in an increase of the fraction of propidium iodide-positive cells (an indication of bacterial death due to compromised membrane integrity) that was not observed when the cultures were added with an equivalent amount of NaCl. Based on these results, it may be that fluorometabolite leakage from a fraction of the engineered bacterial population could account (at least partially) for the presence of 5′-FDRP in culture supernatants. No fluorometabolite could be measured before NaF addition to the cultures, whereas the concentration of 5′-FDRP increased over time in cultures of *P*. *putida* KT2440::FRS-T7RNAP bearing either *P*_*T7*_ → *flA*^*S*. *xinghaiensis*^/*flB1*_His_^*Streptomyces* sp. MA37^ or *flA1* and *flB1*_His_ from strain MA37 to yield similar fluorometabolite outputs (Fig. 5b). When normalized to the biomass in these cultures, the 5′-FDRP concentration ranged between 0.1 and 0.2 μg mg_CDW_^−1^. In contrast with results obtained in vitro, *P*. *putida* KT2440::FRS-T7RNAP expressing the genes encoding FlA and FlB from *S*. *xinghaiensis* produced a very low (but a consistently detectable) amount of 5′-FDRP, consistent with slower PNP kinetics than the other variants tested (which would require prolonged incubation for the biosynthesis of fluorosugars).

## Discussion

Nature has used halogenation widely for tuning the properties of secondary metabolites—but mostly through the introduction of chlorine and bromine atoms^39,40^, as reflected by the rich and diverse enzymology for such bio-halogenations that continues to be revealed and is being harnessed for biotechnology^41^. However, this state of affairs hardly extends to fluorination. In vivo biosynthesis of organofluorine metabolites presents a challenge to biochemistry due to the particular properties of the F^−^ anion, which is a very poor nucleophile in water, and because F has the highest oxidation potential of all of the halides, and is not oxidized (to fluoronium, F^+^) unlike the other halogens. These properties severely limit the potential for the creation of C–F bonds from F^−^ by either nucleophilic or electrophilic processes, and only the nucleophilic fluorinase enzyme is known so far from Nature. In this study, we have contributed to address the biotechnological challenge of site-specific and stereo-selective fluorination by designing synthetic gene circuits that trigger biofluorination in response to inorganic F^−^, the basal substrate of the engineered pathway. By pairing a F^−^-binding *cis*-regulatory RNA with the orthogonal T7 RNA polymerase, the transcription of FluoroBricks encoding fluorinases and PNP enzymes was activated by the addition of small amounts of NaF to cultures of engineered *P*. *putida*—leading to the formation of fluoronucleotides and fluorosugars both in vitro and in vivo.

The FluoroBrick standard proposed here enabled the fast combination of different functional modules, a feature that can be expanded by adding other *bricks* encoding further enzymatic activities. The system has been tailored for *P*. *putida*, but it could easily be transplanted into other Gram-negative bacterial species^42^, e.g., by adopting the Standard European Vector Architecture^43^. We have prototyped the synthetic circuits in cell-free extracts of the same bacterial host to be used for in vivo assays, and the use of FluoroBricks enabled organofluorine biosynthesis from inorganic F^−^ (i.e. de novo biofluorination) without the typical requirement for priming the cells with advanced organofluorine supplements. Importantly, 5′-FDA (and 5′-FDRP) was formed from endogenous SAM in the cells. Considering that the intracellular concentration of SAM in Gram-negative bacteria is typically within the 100–200 μM range^44^, further improvements in fluorometabolite biosynthesis can be anticipated by increasing precursor availability, e.g., as demonstrated in *E*. *coli* strains engineered for the production of *O*-methylated anthocyanin^45^. At the same time, the dual FRS-T7RNAP/*P*_*T7*_ → *msfGFP* system can be adapted as a biosensor of the intracellular F^−^ concentration in vivo; a feature recently exploited for F^−^ detection in a cell-free system for point-of-use detection of F^−^ in water^46^. Although this dual system could not enable efficient biofluorination in a Δ*crcB* mutant of *P*. *putida*, this combination of strain and synthetic gene circuit constitutes a valuable system to trigger gene expression when F^−^ is not a substrate of the target biochemical pathway. In such a case, minute amounts of NaF could be used to efficiently activate the system with an optimal dose–response behavior.

*P*. *putida* tolerated NaF concentrations that typically inhibit the growth of both natural fluorometabolite producers^47^ and surrogate bacteria engineered for synthesis of organofluorines^16^. Furthermore, the onset of fluorometabolite biosynthesis in *S*. *cattleya* takes place after 6 days of cultivation in the presence of 2 mM NaF, and maximum titers of final products (i.e. fluoroacetate and 4-fluorothreonine) are reached only after 28 days of incubation^47^. The engineered *P*. *putida* strains presented in this study produced both fluoronucleotides and fluorosugars after a 48-h cultivation in a minimal medium with added glucose and NaF as the only carbon and fluorine sources, respectively. Interestingly, 5′-FDA was not converted to 5′-FDRP (or any other fluorinated derivative) in the absence of a FluoroBrick-encoded PNP, indicating that the endogenous phosphorylase activities of strain KT2440 are largely unreactive toward 5′-FDA. This circumstance can be exploited for the design of *neo*-metabolism^48^, where metabolic nodes based on fluorometabolites, orthogonal to the extant biochemistry of the host, are exploited to drive biosynthesis of organofluorines (e.g. fluoro-amino acids, which can be incorporated into proteins^49,50^). Taken together, our results show how the manipulation of synthetic circuits, together with the adoption of a robust platform strain, can be harnessed for the biosynthesis of complex molecules beyond the customary products of cell metabolism.

## Methods

### Bacterial strains and growth conditions

All bacterial strains and plasmids used in this study are listed in Supplementary Table 1. *Pseudomonas putida* KT2440 was routinely grown at 30 °C in LB medium^51^ or LB medium agar plates. A modified M9 minimal medium^52^ containing 5 g L^−1^ glucose was used for all expression experiments and fluorometabolite biosynthesis assays. *E. coli* DH5α was used as host for cloning and plasmid maintenance. *E*. *coli* was cultured in LB medium at 37 °C. Antibiotics were added when needed as follows: chloramphenicol, 30 μg mL^−1^; kanamycin, 50 μg mL^−1^; gentamicin, 10 μg mL^−1^; and streptomycin, 100 μg mL^−1^. Electrocompetent *P. putida* cells were prepared by washing the biomass with 300 mM sucrose^53^. NaF was purchased from Sigma-Aldrich Co. (St. Louis, MO, USA) and used as inducer, substrate for biofluorination reactions and stressor as indicated in the main text; 3-methylbenzoate was used for induction of the XylS/*Pm* expression system at 1 mM^38^.

### Construction of recombinant plasmids and engineered strains

Four versions of the *P. syringae* riboswitch, FRSv1-4, were synthesized by Integrated DNA Technologies Inc. (Coralville, IA, USA) and cloned into plasmid pSEVA441 by *USER* cloning^54^, using primers specified on Supplementary Table 2, together with the *msfGFP* gene, which was separately amplified from vector pSEVA427. Fragments were amplified using uracil-containing primers, digested with *Dpn*I and 100 ng of each amplicon was mixed with the USER enzyme in a final volume of 10 μL. After incubating at 37, 24, and 10 °C (for 15 min each step), 5 μL of the mix was transformed into *E*. *coli* DH5α and cells were plated onto LB medium agar added with the corresponding antibiotics, which yielded plasmids pS441·FRSv1, pS441·FRSv2, pS441·FRSv3, and pS441·FRSv4. Plasmid pTn7::FRS·T7RNAP was constructed by translationally fusing the synthetic FRSv1 fragment, containing the riboswitch together with the original promoter and the triplets encoding the first seven amino acids of EriC^F^ from *P*. *syringae*, to the gene encoding T7 RNA polymerase with primers indicated in Supplementary Table 2. The integration of the FRS-regulated T7 RNA polymerase gene into the chromosome was achieved by electroporating plasmid pTn7::FRS·T7RNAP together with helper plasmid pTNS2 into freshly prepared *P. putida* KT2440 and *P. putida* KT2440Δ*crcB* electrocompetent cells, followed by colony PCR checking and DNA sequencing^55^. All relevant sequences are listed in Supplementary Data 1.

Gene deletions in *P*. *putida* KT2440 were performed by CRISPR/Cas9-assisted genome engineering^56^ using DNA sequences taken from the latest annotation of the genome of strain KT2440^32^. Plasmid pSEVA231-CRISPR*crcB* was constructed for the deletion of *crcB*. First, oligonucleotides *crcB*-CRISPR and *crcB*-CRISPR-R were phosphorylated by incubating 1 μL of each primer suspension at 100 μM with the enzyme T4 PNK and annealed together by incubating 30 min at 37 °C, 4 min at 95 °C and finally decreasing the temperature 5 °C per min until 25 °C were reached. The annealed primers were ligated with plasmid pSEVA231-CRISPR, previously digested with *Bsa*I, using T4 ligase. The ligation was transformed by heat-shock into *E*. *coli* DH5α cells and plated onto LB medium agar plates with the corresponding antibiotics and incubated at 37 °C overnight. The correct constructions were checked by colony PCR and DNA sequencing. To delete *crcB*, an overnight culture of *P*. *putida* KT2440-containing plasmids pSEVA658::SSR and pSEVA421::Cas9-tracrRNA was diluted to an optical density measured at 600 nm (OD_600_) of 0.1, and grown until the OD_600_ reached 0.4–0.5 units. Cells were subsequently induced to express the gene encoding the SSR recombinase with 1 mM of 3-methylbenzoate for 3 h at 30 °C. After induction, electrocompetent *P. putida* KT2440 cells were electroporated using 100 ng of pSEVA231-CRISPR::*crcB* plasmid and 1 μL of a 100 μM solution of primer *crcB*del-Rec, a recombineering oligonucleotide containing 45 bp of the upstream and downstream region including the *ATG* and *STOP* codons of *crcB* for its elimination. Cells were recovered in LB medium at 30 °C for 2 h, plated onto LB medium agar plates with the antibiotics needed and incubated overnight at 30 °C. The successful recombineering event was checked by colony PCR and DNA sequencing. Isolated *P. putida* KT2440Δ*crcB* clones were cured from the helper plasmids by serial dilution under non-selective conditions.

Modules used for biofluorination are based on *flA1* and *flB1* from *Streptomyces* sp. MA37 and *SxflA* and *SxflB* from *S*. *xinghaiensis*, codon-optimized and synthesized by GeneCust Europe (Boynes, France) to yield *FluoroBricks* that can be cloned in different configurations using standard procedures (Supplementary Fig. 1). Plasmids pFB·2F1 and pFB·1F1 were constructed by cloning *flA1* into vectors pPS23 or pS231T7, containing the XylS/*Pm* or P_T7_ expression systems, respectively, using the restriction sites *Avr*II and *Eco*RI. In parallel, plasmids pFB·2F2 and pFB·1F2 were constructed by cloning *SxflA* using the same procedure. Plasmids pFB·1F1P^H^1 and pFB·1F2P^H^2 were obtained by amplifying synthetic genes encoding either FlB phosphorylase with USER primers containing a 6× His tag immediately after the start *ATG* codon in the forward primer. A two-fragment USER cloning was performed to combine *flA* and *flB* variants in different configurations as indicated in the text.

### Construction of phylogenetic trees

CrcB protein sequences were obtained from the Uniprot database^57^ (https://www.uniprot.org/), selecting reviewed and properly annotated amino acid sequences from different *Pseudomonas* species. A multiple sequence alignment was performed using ClustalW^58^, with a subsequent phylogenetic analysis using the PhyML 3.0 software^59^.

### Characterization of the fluoride-responsive riboswitch

Overnight pre-cultures of *P. putida* KT2440 transformed with plasmids pSEVA441 (auto-fluorescence control) or pS441·FRSv1-FRSv4 were diluted 40 times in 10 mL of modified M9 minimal medium, and distributed in 96-well microtiter plates (flat bottom; Greiner Bio-One, Kremsmünster, Austria). Cells were grown for 5 h at 30 °C and 300 rpm, after which the cultures were added with NaF at 1, 5, and 15 mM. Kinetics of growth (OD_600_) and fluorescence, using wavelengths of excitation and emission of 485 nm and 528 nm, respectively, were performed using a Synergy^TM^ MX microtiter plate reader (BioTek Instruments Inc., Winooski, VT, USA) for 20 h. Measurements were carried out every 10 min in three independent biological replicates. *P. putida* KT2440::FRS·T7RNAP and *P. putida* KT2440Δ*crcB*::FRS·T7RNAP carrying either plasmid pS231·PT7::*msfGFP* or vector pS231·PT7, used as an auto-fluorescence control, were incubated in 96-deep well plates. In this case, cells were grown for 5 h at 30 °C and 300 rpm, after which the cultures were added with NaF at 1, 2.5, 5, 7.5, 10, 12, and 15 mM (*P. putida* KT2440::FRS·T7RNAP) or 0.1, 0.25, 0.5, and 1 mM (*P. putida* KT2440Δ*crcB*::FRS·T7RNAP).

### Fluoride toxicity assays

Overnight cultures of *P. putida* KT2440 and *P. putida* KT2440Δ*crcB* were diluted 20 times in 10 mL of modified M9 minimal medium containing different concentrations of NaF. Cells were grown in 96-well microtiter plates (flat bottom, Greiner Bio-One) at 30 °C and 200 rpm in an ELx808 microtiter plate reader (BioTek Instruments Inc.). Cells exposed to NaF at various concentrations were incubated in a microtiter plate reader for 24 h, and growth was followed by measuring OD_630_ every 30 min. OD_630_ values after 20 h were then selected to plot bacterial growth as a function of the F^−^ concentration. The NaF concentrations used for each strain were 0, 25, 50, 75, 125, 200, and 250 mM for *P. putida* KT2440, and 0, 0.1, 0.5, 1 and 5 mM for *P. putida* KT2440Δ*crcB*. Drops assays were performed using overnight cultures of strains *P. putida* KT2440 and *P. putida* KT2440Δ*crcB* in modified M9 minimal medium. These cultures were serially diluted in fresh medium and 10 μL of the dilutions were spotted onto modified M9 minimal medium agar plates supplemented with NaF at 0, 1, or 10 mM. Droplets were let dry out before placing the plates at 30 °C for 16 h.

### Biosynthesis of fluorometabolites in vitro and in vivo

In vitro biofluorination assays were performed using overnight cultures of the strains indicated in the text and diluting them to an OD_600_ of 0.1 in 50 mL of fresh modified M9 minimal medium with the appropriate antibiotics in 250-mL shake flasks. Cells were grown at 30 °C with shaking of 180 rpm until they reached an OD_600_ of 0.4–0.6, after which the expression of the relevant FluoroBricks was induced with 1 mM 3-methylbenzoate (for the XylS/*Pm* system) or 15 mM NaF (for the dual FRS-T7RNAP/*P*_*T7*_ → *msfGFP* system). After 20 h of incubation, cells were harvested by centrifugation at 4500 × *g* for 10 min at 4 °C. Cellular pellets were washed with 50 mM Tris·HCl pH = 7.8 and re-suspended in a final volume of 3 mL. Cells were disrupted using glass beads in a cell homogenizer (Precellys 24; Bertin instruments, Montigny-le-Bretonneux, France), using a program of 6000 rpm for 20 s. The suspension was centrifuged at 17,000 × *g* for 2 min at 4 °C, and the supernatant was transferred to a new tube. Protein concentration was determined using a Bradford assay^60^, consistently yielding a total protein concentration of at least 1 mg mL^−1^. The cell-free extract (1 mL) was mixed with 0.2 mM SAM and 5 mM NaF in 50 mM Tris·HCl pH = 7.8. This reaction mixture was incubated for 20 h at 30 °C, and inactivated by boiling the samples at 95 °C for 5 min and centrifuging at 17,000 × *g* for 10 min. The supernatant was analyzed for the presence of fluorometabolites by LC–MS as indicated below.

The same procedure was scaled up to yield enough amount of fluorometabolites to be detected by ^19^F-NMR. To this end, 500 mL of fresh modified M9 minimal medium with the appropriate antibiotics was inoculated with overnight cultures in 2-L Erlenmeyer flasks, cultures were grown to an OD_600_ of 0.4–0.6 and then the expression systems were induced as indicated. After 24 h of induction, cells were harvested by centrifugation at 4700 × *g* for 20 min at 4 °C, and re-suspended in 35 mL of 50 mM Tris·HCl pH = 7.8. Cells were subsequently treated with a French press (Avestin Emulsiflex C5; ATA Scientific Instruments, Taren Point, Australia) to break them open, and the lysates were centrifuged at 10,000 × *g* for 20 min. Supernatants (10 mL, containing the same amount of total protein across assays) were used directly for biofluorination reactions by mixing them with 0.2 mM of SAM and 5 mM of NaF (in 50 mM Tris·HCl pH = 7.8) in a final reaction volume of 20 mL. This reaction was incubated for 20 h at 30 °C, and then inactivated by boiling the samples at 95 °C for 5 min and centrifuging the reactions at 17,000 × *g* for 10 min. Finally, 100-μL aliquots of the supernatant were taken and analyzed by LC–MS, and the remaining sample was submitted to ^19^F-NMR analysis (see details below).

In vivo biofluorination assays were carried out with *P. putida* KT2440::FRS·T7RNAP harboring plasmids containing *flA* and *flB* from different *Streptomyces* as indicated in the text. Overnight cultures were diluted to an OD_600_ of 0.1 in 50 mL of fresh modified M9 minimal medium in 250-mL Erlenmeyer flasks. Cells were grown at 30 °C with shaking of 180 rpm until they reached an OD_600_ between 0.4 and 0.6 and gene expression was induced with 10 mM NaF. For experiments with *P. putida* KT2440Δ*crcB*::FRS·T7RNAP, NaF was added at 5 mM. After incubating the cultures for 2, 20, 24, and 48 h post induction, OD_600_ was measured and the CDW of the samples were calculated as indicated elsewhere^61^. At each data point, 2-mL samples were also retrieved for fluorometabolite extraction and determination. The same strain carrying the corresponding empty vector was used as negative control. Three independent biological replicates were carried out for each condition.

For metabolite extraction from the bacterial pellets, cells were centrifuged at 5000 × *g* for 10 min, and the supernatants and pellets were separated. Cells were washed once with 50 mM phosphate buffer and incubated with 0.5 mL of extraction solution^62^ [60% (v/v) ethanol, 10 mM ammonium acetate pH = 7.2] at 78 °C for 1 min. Cell suspensions were subsequently centrifuged at maximum speed for 1 min and the supernatant was placed in a new Eppendorf tube. This extraction step was repeated three times, the extracts were pooled and then evaporated in a SpeedVac concentrator (Thermo-Fisher Scientific Co., Waltham, MA, USA) for at least 6 h. Pellets were eluted in 100 μL of milli-Q water and analyzed in a LC–MS system as explained below.

### Dead and alive bacterial assay

The membrane integrity of *P*. *putida* cells incubated in the presence of NaF was tested using propidium iodide^63^. Engineered and wild-type *P*. *putida* strains were grown as explained above for in vivo production of fluorometabolites, adding NaF at 10 mM to some of the cultures when the OD_600_ reached 0.4–0.6. After 24 h of incubation, samples were harvested and diluted with the same volume of fresh minimal medium. A 0.2-mL aliquot of the cell suspension was then transferred to a 96-well-plate and incubated with propidium iodide to a final concentration of 12.5 μg L^−1^ for 5 min with orbital shaking. The OD_600_ (i.e.. bacterial growth) and the fluorescence at excitation/emission wavelengths of 544/612 nm (representing the fraction of dead cells) were detected using a Synergy^TM^ MX microtiter plate reader (BioTek Instruments Inc.).

### LC–MS analysis of fluorometabolites

Fluorine-containing metabolites were analyzed using a Prominence XR (Shimadzu, Columbia, MD, USA) HPLC system coupled to a 5500 QTRAP mass spectrometer (Sciex, Framingham, MA, USA). The auto-sampler was cooled to 15 °C, and the metabolites in 10-μL injections were separated on an XSelect HSS T3 150 × 2.1 mm^2^ × 2.5 μm column (Waters, Milford, MA, USA). The column temperature was held at 40 °C, and the metabolites were eluted with a constant flow rate of 0.4 mL min^−1^. The elution profile began with 100% buffer A [10 mM tributylamine, 10 mM acetic acid (pH = 6.86), 5% (v/v) methanol and 2% (v/v) 2-propanol]. Starting at 4 min, the amount of buffer B (2-propanol) was linearly increased to 15% at 8 min and hold at 15% until 12 min. Buffer B was then linearly decreased to 0% until 13.5 min and kept at 0% until 17 min. The mass spectrometer was operated in negative mode with multiple reaction monitoring, and unit resolution for the mass filter Q1 and Q3 was used for detection and quantification. The parameters for electrospray ionization were as following: electrospray voltage −4500 V, temperature 500 °C, curtain gas 40 psi, CAD gas 12 psi, and gas 1 and 2 each 50 psi and collision gas high. Detection parameters were optimized for each fluorometabolite (Supplementary Table 3). For each compound, a separate transition for quantification and for qualification was used. Authentic 5′-FDA and 5′-FDRP standards were used as references and for preparing calibration curves for quantification (see details on synthesis below). The Prism 8 software (GraphPad Software Inc., San Diego, CA, USA) was used to plot peaks obtained for selected fluorometabolites.

### Chemical synthesis, purification, and characterization of 5′-FDA

The chemical synthesis of 5′-FDA was performed as indicated in Supplementary Fig. 3. For the last synthetic step, 2,2,2-trifluoroacetic acid (TFA) is conventionally used in the deprotection of the 2′,3′-isopropylidene group^64^. This treatment results in a product that is a TFA salt (and therefore interferes with the detection of other fluorometabolites by ^19^F-NMR analysis). To avoid having TFA in the product, other deprotection strategies were examined. Using 90% (v/v) formic acid turned out to be the best approach achieving the desired product in good yield (80–85% mol/mol). Thus, in the last step of our synthesis approach, 2′,3′-isopropylidenated 5′-FDA (0.49 g, 1.58 mmol) was dissolved in 10 ml of 90% (v/v) formic acid for 3-4 h at room temperature and the solution was evaporated to dryness. The solid was purified by flash chromatography (ethyl acetate/CH_3_OH = 9:1) to give 5′-FDA as a white solid (0.36 g, yield = 84% mol/mol). Flash column chromatography was performed with this material using Geduran Si 60 (40–63 μm) silica gel (Merck KGaA, Darmstadt, Germany). A thorough characterization of the product was performed by ^1^H- and ^19^F-NMR. NMR data were acquired at 298 K using either a 600 MHz Bruker AVANCE III HD spectrometer equipped with a Bruker BBFO SmartProbe or a 800 MHz Bruker AVANCE III HD spectrometer equipped with a TCI Cryoprobe. Chemical shifts (δ) are reported in parts per million (ppm) and coupling constants in (*J*) in Hz. For spectra acquired in D_2_O, chemical shifts are reported relative to the signal for HDO (δ = 4.79 ppm for ^1^H-NMR) and ^13^C chemical shifts are referenced using the deuterium lock-signal from solvent with δ [Si(CH_3_)_4_] = 0 ppm.

### Enzymatic synthesis of 5′-FDRP

5′-FDRP was enzymatically synthesized using one unit of a commercial PNP enzyme (N2415, Sigma-Aldrich Co.) and purified 5′-FDA (4 mM) as the substrate in 50 mM K_2_HPO_4_ (pH = 7.5). The reaction was incubated at 30 °C for 15 h and stopped by heat inactivation of the enzyme at 95 °C for 5 min. A transformation yield of 5′-FDA into 5′-FDRP around 40–50% (mol/mol) was consistently achieved under these conditions. The fluorosugar product was characterized by ^19^F-NMR as indicated above, and serial dilutions of the reaction mixture was used to prepare the calibration curve for LC–MS measurements of 5′-FDRP.

### ^19^F-NMR analysis

Biological samples were lyophilized from a frozen solution in water using a Christ Alpha 1–2 LD Plus freeze drier (Martin Christ Gefriertrocknungsanlagen GmbH, Osterode am Harz, Germany). To the freeze-dried sample was added an MeOD/D_2_O solution, and the resulting suspension was subjected to sonication to ensure dissolution of the fluorometabolites present in the sample. ^19^F-NMR experiments were then recorded at 298 K on a Bruker AVANCE III HD instrument with either a SmartProbe BBFO+ (for proton coupled experiments) or TCI CryoProbe (for proton decoupled experiments), using CFCl_3_ as an external reference^13^. Chemical shifts are reported in ppm as indicated above.

### Statistical analysis

Data analysis has been done with MS Excel unless otherwise stated. All numerical values are depicted as means ± standard deviations. Statistical differences in fluorometabolite biosynthesis between engineered and modified strains in key experiments were assessed via a two-tailed Student’s *t* test assuming equal variances using Prism 8 (GraphPad Software Inc.). In all cases, *p* values < 0.05 were considered significant.

### Reporting summary

Further information on research design is available in the Nature Research Reporting Summary linked to this article.

## Supplementary information

Supplementary Information

Peer Review File

Reporting Summary

Description of Additional Supplementary Files

Supplementary Data 1

## Data Availability

Data supporting the findings of this work are available within the paper and its Supplementary information files. A reporting summary for this article is available as a Supplementary Information file. The datasets generated and analyzed during the current study are available from the corresponding author upon request. Source data are provided with this paper.

## Source data

Source Data
