# Supplementary material for: A fluoride-responsive genetic circuit enables in vivo biofluorination in engineered Pseudomonas putida
Source: Nat Commun. 2020 Oct 7;11:5045. doi: 10.1038/s41467-020-18813-x (PMC7541441; doi:10.1038/s41467-020-18813-x)
Supplement: Supplementary file 1 — Supplementary Information [file 41467_2020_18813_MOESM1_ESM.pdf]

A fluoride-responsive genetic circuit enables *in vivo* biofluorination in engineered *Pseudomonas putida*

Calero *et al.*

Supplementary Table 1. Bacterial strains and plasmids used in this study.

Strains	Relevant characteristics [a]	Reference
E. coli DH5α	Cloning host; F^{-} λ^{-} *endA1 glnX44*(AS) *thiE1 recA1 relA1 spoT1 gyrA96*(Nal^{R}) *rfbC1 deoR nupG* Φ80(*lacZ*Δ*M15*) Δ(*argF-lac*)*U169 hsdR17*(r_K^- m_K^+)	Meselson and Yuan[1]
P. putida KT2440	Wild-type strain; derivative of *P. putida* mt-2 cured of the TOL plasmid pWW0, *hsdR1*(r^- m^+)	Bagdasarian *et al.*[2]
P. putida KT2440Δ*crcB*	*P. putida* KT2440 derivative, in-frame deletion of *crcB* (*PP_4001*)	This study
P. putida KT2440 ::FRS-T7RNAP	*P. putida* KT2440 derivative, mini-Tn*7* module with the gene encoding T7 RNA polymerase under control of FRS integrated after *glmS* (*PP_5409*)	This study
P. putida KT2440Δ*crcB* ::FRS-T7RNAP	*P. putida* KT2440Δ*crcB* derivative, mini-Tn*7* module with the gene encoding T7 RNA polymerase under control of FRS integrated after *glmS* (*PP_5409*)	This study
**Plasmids**	**Relevant characteristics**	**Reference**
pSEVA441	Cloning vector; *oriV*(pRO1600/ColE1); Str^{R}	Silva-Rocha *et al.*[3]
pS441·FRSv1	Derivative of vector pSEVA441, FRSv1→*msfGFP*; Str^{R}	This study
pS441·FRSv2	Derivative of vector pSEVA441, FRSv2→*msfGFP*; Str^{R}	This study
pS441·FRSv3	Derivative of vector pSEVA441, FRSv3→*msfGFP*; Str^{R}	This study
pS441·FRSv4	Derivative of vector pSEVA441, FRSv4→*msfGFP*; Str^{R}	This study
pTn*7*-M	Tn*7* integration vector; *oriV*(R6K); Gm^{R} Km^{R}	Choi *et al.*[4]
pTn*7*::FRS·T7RNAP	Delivery plasmid for mini-Tn*7*[FRSv1→*T7RNAP*] integration; Gm^{R} Km^{R}	This study
pTNS2	Helper plasmid for constitutive expression of *tnsABCD* encoding the Tn7 transposase; *oriV*(R6K); Amp^{R}	Choi *et al.*[4]
pPS23	ProUSER expression vector; *xylS/Pm*, USER cassette; Km^{R}	Calero *et al.*[5]
pFB·2F1	Derivative of vector pPS23, *xylS/Pm*→*flA1*; Km^{R}	This study
pFB·2F2	Derivative of vector pPS23, *xylS/Pm*→*SxflA*; Km^{R}	This study
pSEVA231	Cloning vector; *oriV*(pBBR1); Km^{R}	Silva-Rocha *et al.*[3]
pS231T7	Derivative of vector pSEVA231 containing the T7 promoter; Km^{R}	Volke *et al.*[6]
pFB·1F1	Derivative of vector pS231T7; P_{T7}→*flA1*; Km^{R}	This study
pFB·1F2	Derivative of vector pS231T7; P_{T7}→*SxflA*; Km^{R}	This study
pFB·1F1P^{H}1	Derivative of vector pS231T7; P_{T7}→*flA1-NHisflB1*; Km^{R}	This study
pFB·1F2P^{H}2	Derivative of vector pS231T7; P_{T7}→*SxflA-NHisSxflB*; Km^{R}	This study
pFB·1F2P^{H}1	Derivative of vector pS231T7; P_{T7}→*SxflA-NHisflB1*; Km^{R}	This study
pFB·2F21P^{H}1	Derivative of vector pPS23, *xylS/Pm* →*flA1-NHisflB1*; Km^{R}	This study
pFB·2F2P2	Derivative of vector pPS23, *xylS/Pm* →*SxflA-SxflB*; Km^{R}	This study
pS231T7::msfGFP	Derivative of vector pS231T7; P_{T7}→*msfgfp*; Km^{R}	This study
pSEVA231-CRISPR	Helper plasmid for genome engineering; *oriV*(pBBR1); CRISPR array; Km^{R}	Aparicio *et al.*[7]
pSEVA231-CRISPR::*crcB*	Derivative of vector pS231-CRISPR with a target region for *crcB*	This study
pSEVA658-*ssr*	Helper plasmid for genome engineering; *xylS/Pm*→*ssr*; *oriV*(RFS1010); Gm^{R}	Aparicio *et al.*[7]
pSEVA421-Cas9tr	Helper plasmid for genome engineering; *cas9*; *oriV*(RK2); Str^{R}	Aparicio *et al.*[7]

[a] *FRS*, fluoride-responsive riboswitch; *Antibiotic markers*: Amp, ampicillin; Gm, gentamicin; Km, kanamycin; Nal, nalidixic acid; and Str, streptomycin.

Supplementary Table 2. Oligonucleotides used in this study.

Name	Sequence (5'→3')	Purpose
*flA1*_F_*Avr*II<br>*flA1*_R_*Eco*RI	TCGACCTAGGAGGAGGAAAAACATATGGCC<br>TCGAGAATTCTCAACGGGCCTCC	*flA1* amplification for cloning using *Avr*II and *Eco*RI sites
Sxin*flA**-U-F<br>Sxin*flA**-U-R	AGGCGAUAGGAGGAGGAAAAACATATGTCCGCCG<br>AGGTGCGAUTCAGTTGGTTTCAACGCGCACCTTGAG	*SxflA* amplification for USER cloning in pPS23
PT7Sxin*flA*_U_F<br>PT7Sxin*flA*_U_R	ATCCTCUAGGAGGAGGAAAAACATATGTCCGCCG<br>AGCTTGCAUTCAGTTGGTTTCAACGCGCACCTTGAG	*SxflA* amplification for USER cloning in pS231T7
*flB1*NHis-U-F<br>*flB1*-U-R	ATGCAAGCUAGGAGGAGGAAAAACATATGCATCATCACC<br>AGGCGAUTCACACGACCGGGGCGATG	USER primers to clone *flB1*-NHis into pFB·PT7flA1
pSEVA_U_*flB*<br>*flA1*_RSv1_U_R	ATCGCCUTGCGGCCGCGTCGTGACTG<br>AGCTTGCAUTCAACGGGCCTCCACGCGC	USER primers to amplify vector pFB·PT7flA1
Sx*flB*-Histag-F<br><br>Sx*flB*-Histag-R	ACATGCAUCATCACCACCACCATAGAGAAACCAGAGGCG<br>CAGCATTCC<br>ATGCATGUGTTTTTCCTCCTCTCGAGGAA	*SxflB*-NHis amplification for USER cloning in pFB·PT7SxflA
pTn7-U-R<br>pTn7-U-F	AGAGGAUTTAATTAAGACGTCTTGACATAAGCC<br>ACGACCUAACACGATTAACATCGCTAAGAAC	USER primers to amplify backbone pTn7-M
PsyrProm_U_F	ATCCTCUTTTGGCCCTCTTTCGTAAG	USER primer to amplify FRSv1 and FRSv2
Rib_EriC_U_R	ACTAGGUCGTCGAAATTTAGACATT	USER primer to amplify FRS1 and FRSv3
PEM7_U_F	ATCCTCUTTGTTGACAATTAATCATCG	USER primer to amplify FRSv3 and FRSv4
Rib_ATG_U_R	ACTCATUTGGCCAGCCACAAAAAATAAG	USER primer to amplify FRSv2 and FRSv4
GFPEriC_U_F	ACCTAGUAAAGGAGAAGAACTTTTC	USER primer to amplify *msfGFP* in FRSv1 and FRSv3
GFP_ATG_U_F	AATGAGUAAAGGAGAAGAACTTTTC	USER primer to amplify *msfGFP* in FRSv2 and FRSv4
GFP_U_R	AGCTTGCAUCTATTTGTATAGTTCATCC	USER primer to amplify *msfGFP*
pSEVA_U_F<br>pSEVA_U_R	ATGCAAGCUTGCGGCCGCGTC<br>AGAGGAUCCCCGGGTACCGAGC	USER primers to amplify pSEVA441 and pS231T7
RSv1-T7RNApol-U-R	AGGTCGUCGAAATTTAGACATTTGG	USER primer to amplify FRSv1
crcB-CRISPR<br>*crcB*-CRISPR-R	AAACTTTCCATCAGGCGCACGGTATCGAGCGAGAG<br>AAAACTCTCGCTCGATACCGTGCGCCTGATGGAAA	Primers for gRNA synthesis and cloning into pSEVA231-CRISPR
*crcB*del-Rec	GGAATCGAGCATATCGTTCTCTCGTTATATGTAGGTGTTG<br>GTTCACATGGTTTTCTCCGCTGGCGGGGGCTGTTGCGGT<br>CGAGGTCAGCC	Recombineering oligo for *crcB* deletion in strain KT2440

Supplementary Table 3. Transitions and optimized parameters for fluorometabolites detection by mass spectrometry.[a]

Compound	Parent ion	Parent mass (m/z)	Probable product formula (Hill notation)	Product mass (m/z)	DP (V)	EP (V)	CE (V)	CXP (V)	RT (min)
5'-FDA	$C_{10}H_{11}FN_5O_3^-$	269	$C_5H_7FO_3^-$	134	–65	–10	–64	–21	2.43
	$C_{10}H_{11}FN_5O_3^-$	269	$C_4H_8FO_2^-$	107	–65	–10	–14	–19	2.43
5'-FDRP	$C_5H_9FO_7P^-$	231	HPO_3^-	79	–40	–10	–24	–7	7.5
	$C_5H_9FO_7P^-$	231	$C_5H_7FO_6P^-$	213	–40	–10	–52	–15	7.5

[a] 5'-FDA, 5'-fluoro-5'-deoxyadenosine; 5'-FDRP, 5'-fluoro-5'-deoxy-D-ribose 1-phosphate; DP, declustering potential; EP, entrance potential; CE, collision energy; CXP, collision exit potential; RT, retention time.

Supplementary Table 4. NMR characterization of chemically-synthesized 5'-fluoro-5'-deoxyadenosine.[a]

5'-Fluoro-5'-deoxyadenosine (5'-FDA)

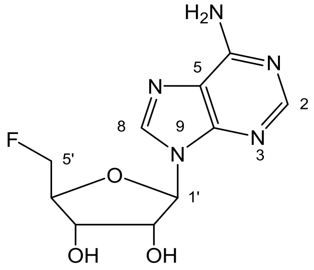

**Atom**	**δ (^{1}H)**	**Int., Mult., *J*** (Hz)	**δ (^{13}C) [Mult., *J*** (Hz)]	**δ$_N$** (^{15}N)	**δ (^{19}F) [Mult., *J*** (Hz)]
1'	6.08	1, d, 5.1	87.4	–	–
2'	4.74	1, m	73.9	–	–
3'	4.54	1, m	69.4	–	–
4'	4.39	1, dm, 29	83.2 (d, 38)	–	–
5'	4.76	2, m	82.5 (d, 165)	–	–231 (td, 48;29)
1	–	–	–	–	–
2	8.25	1, s	151	–	–
3	–	–	–	219.2[b]	–
4	–	–	149	–	–
5	–	–	118.7	–	–
6	–	–	155.5	–	–
7	–	–	–	233.3	–
8	8.32	1, s	140.1	–	–
9	–	–	–	170.4	–

[a] Int., integrals; Mult., multiplicities; s, singlet; d, doublet; t, triplet; m, multiplet.

[b] This assignment is unambiguous and could also be for N_1. Assignments of the NH_2 group are not possible due to exchange of the labile protons.

Note: Both 1D as well as 2D homo- and hetero-nuclear spectra were used to assign all the resonances. The ^{1}H and ^{19}F chemical shifts were obtained from 1D-NMR spectra, whereas the ^{13}C and ^{15}N chemical shifts were obtained from 2D-edited heteronuclear single quantum correlation (HSQC)-, heteronuclear multiple bond correlation (HMBC)- and heteronuclear 2 bond correlation (H2BC)-spectra. The presence of ^{19}F in the molecule enables the extraction of both one and two bond *J* coupling constants between ^{19}F and ^{13}C ($^1J_{FC5'}$ and $^2J_{FC4'}$) from the HSQC spectrum as well as two and three bond *J* coupling constants between ^{19}F and ^{1}H ($^2J_{FH5'}$ and $^3J_{FH4'}$) from the 1D-NMR spectra.

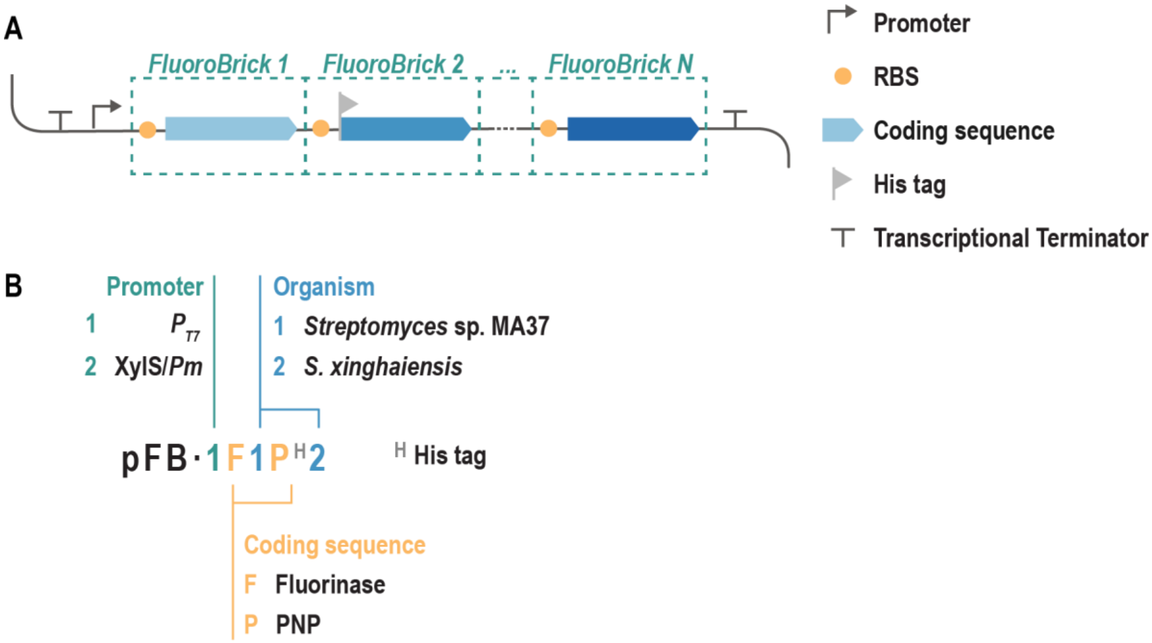

Supplementary Fig. 1. Structural organization of *FluoroBricks*. (A) The standard design followed to test different parts of the biofluorination pathway includes a defined set of synthetic biology parts; i.e. promoters, ribosome binding sites (RBS), coding sequences, His tags and transcriptional terminators. All coding sequences used in this work were codon-optimized to ensure proper expression and translation in *P. putida*. Individual transcriptional units (*FluoroBrick*) are easily composable into more complex designs by one-step *USER* cloning or restriction/ligation according to the rules of the Standard European Vector Architecture[3,8]. **(B)** Nomenclature used for the plasmids containing FluoroBricks. Plasmids are named with the initials *FB* followed by a code of numbers and letters, standing out for the promoter and coding sequence used. An *H* superscript indicates the presence of a 6× histidine tag in the sequence.

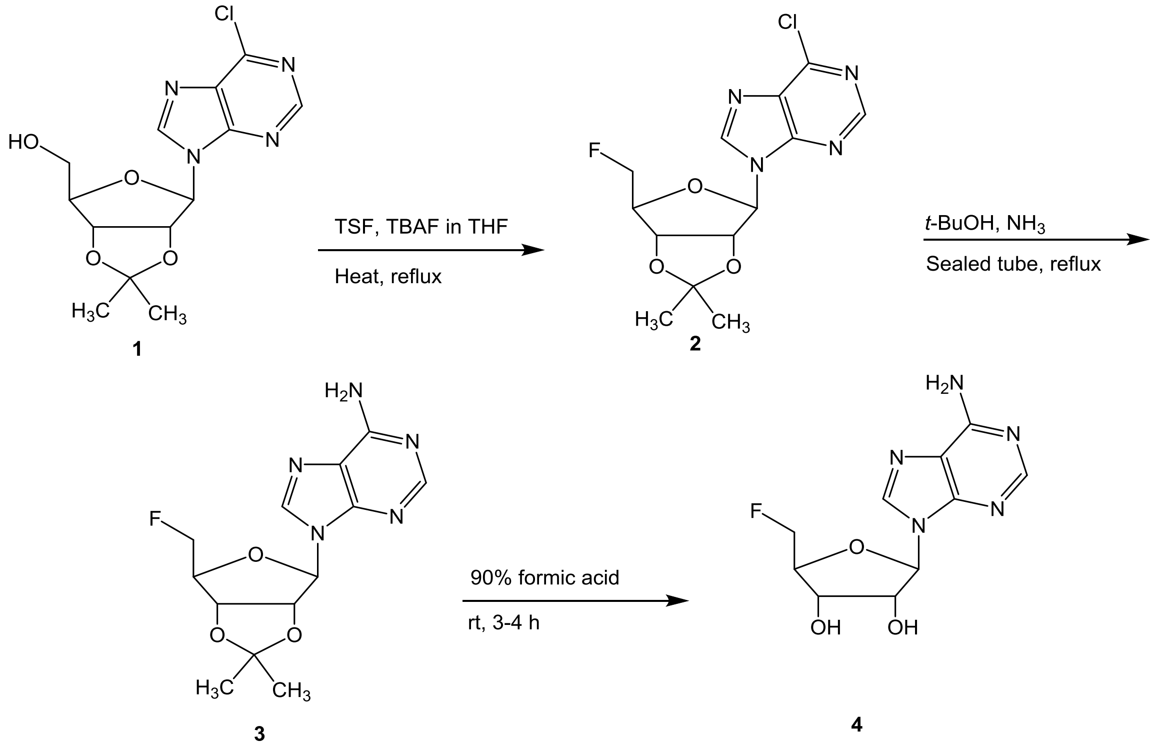

Supplementary Fig. 2. Chemical synthesis of 5'-fluoro-5'-deoxyadenosine. The starting point was 2',3'-isopropylidene-6-chloropurine riboside [6-Chloro-9-(2,3-*O*-isopropylidene-β-D-ribofuranosyl)-9H-purine] (**1**), which was converted to the primary fluoride (**2**) by treatment with tosyl fluoride (TsF) and tetrabutylammonium fluoride (TBAF) in tetrahydrofuran (THF). Next, (**2**) was aminated to 2',3'-isopropylidenated-5'-fluoro-5'-deoxy-adenosine (**3**) with NH_3 in *tert*-butanol (*t*-BuOH). The last step, carried at room temperature (rt), was the deprotection of (**3**) with 90% (v/v) formic acid to yield 5'-fluoro-5'-deoxyadenosine. This protocol is based on the synthesis proposed by Ashton & Scammells[9].

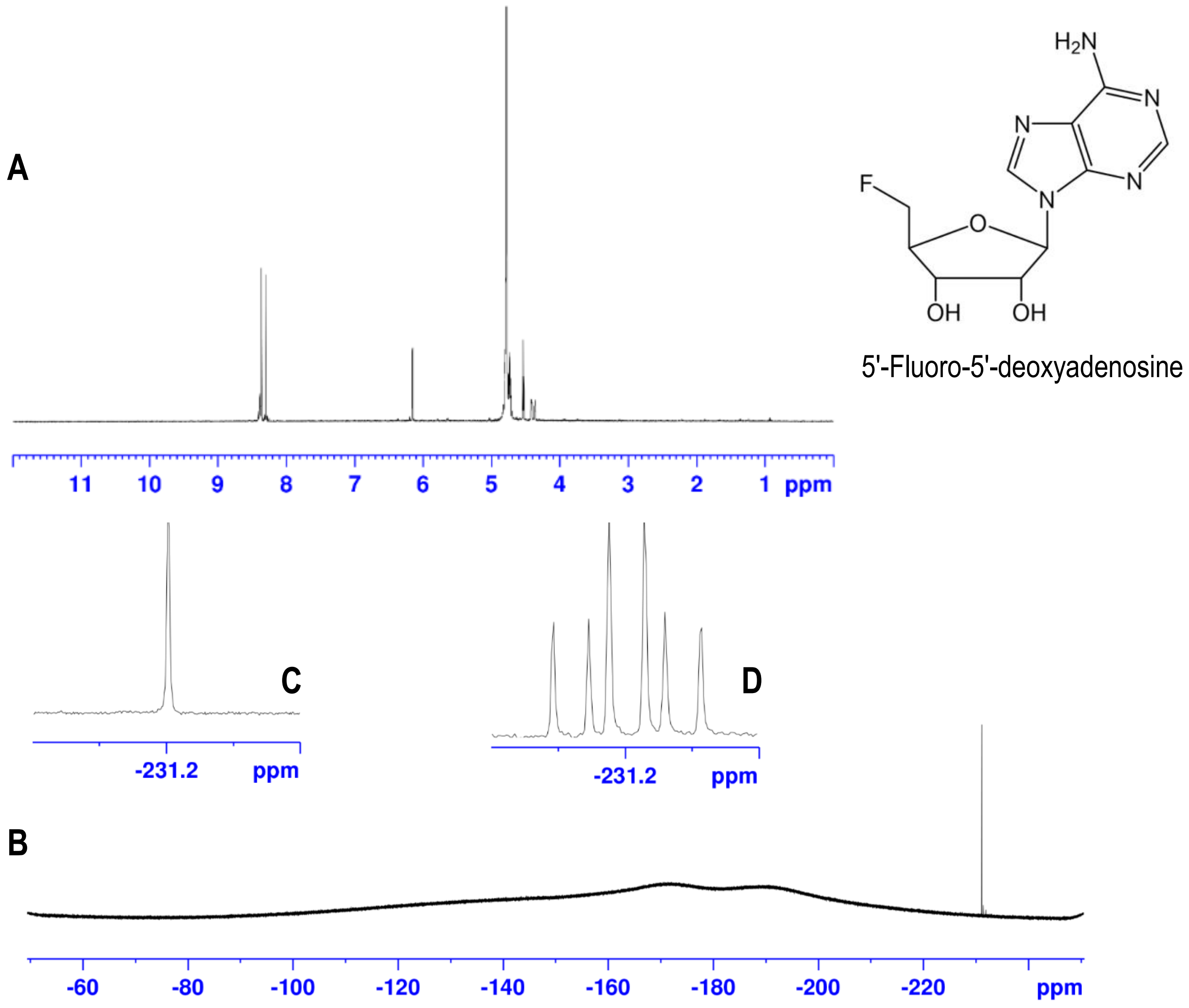

Supplementary Fig. 3. NMR characterization of chemically-synthesized 5'-fluoro-5'-deoxyadenosine. (A) 1D ^{1}H-NMR spectrum of 5'-fluoro-5'-deoxyadenosine (5'-FDA). **(B)** Overview ^{19}F-NMR spectrum. The large hums are background signals originating from the probe. **(C)** Zoom-in of the decoupled ^{19}F-NMR spectrum. **(D)** Zoom-in of the coupled ^{19}F-NMR spectrum.

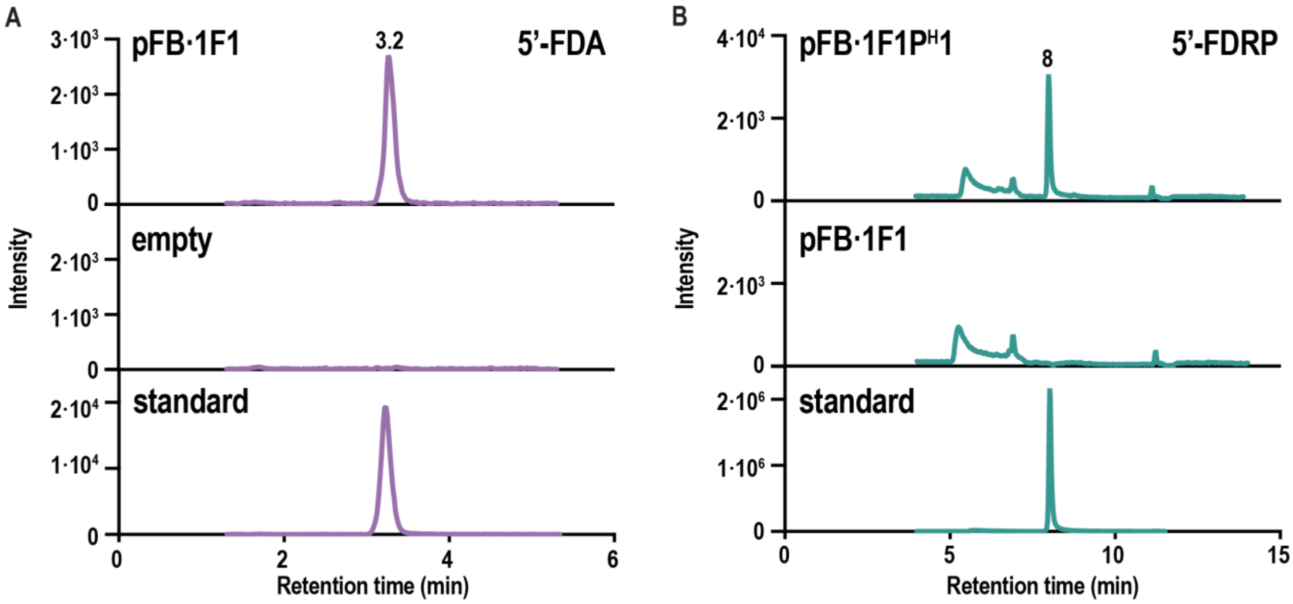

Supplementary Fig. 4. Measurement of fluorometabolites by LC-MS/MS. (A) Chromatograms of cell-free extracts of *P. putida* KT2440 bearing plasmid pFB·1F1 (or an empty plasmid, used as a negative control), compared to the chromatogram obtained for a chemically-synthesized 5'-fluoro-5'-deoxyadenosine (5'-FDA) standard. The characteristic retention time for 5'-FDA (in min) is shown above the peak corresponding to the fluorometabolite. **(B)** Chromatograms of cell-free extracts of *P. putida* KT2440 bearing plasmid pFB·1F1P^{H}1 (or plasmid pFB·1F1, used as a negative control), compared to the chromatogram obtained for an enzymatically-synthesized 5'-fluoro-5'-deoxy-D-ribose 1-phosphate (5'-FDRP) standard. The characteristic retention time for 5'-FDRP (in min) is shown above the peak corresponding to the fluorometabolite. Souce data are provided as a Source Data file.

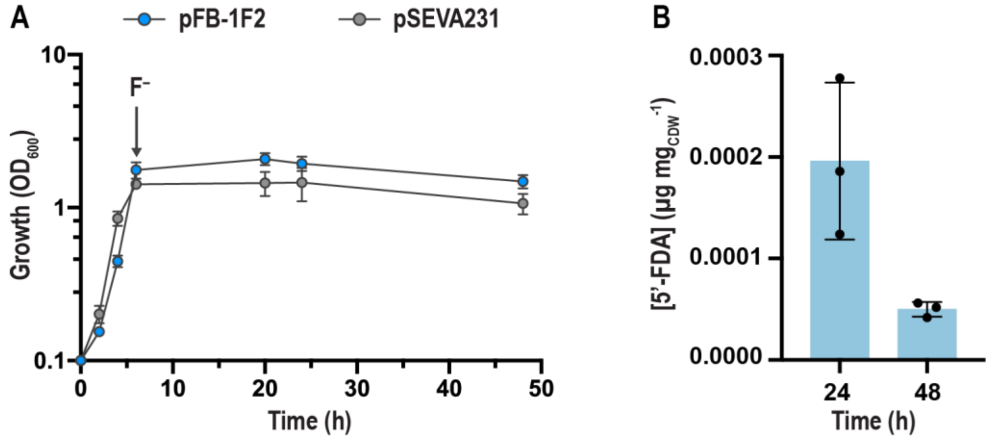

Supplementary Fig. 5. *In vivo* biosynthesis of 5'-fluoro-5'-deoxyadenosine in a Δ*crcB* mutant of *P. putida*. (A) Growth curves of *P. putida* KT2440Δ*crcB*::FRS-T7RNAP containing the plasmid modules with the fluorinase of *S. xinghaiensis* (pFB·1F2, blue circles) compared to the growth of *P. putida* KT2440Δ*crcB*::FRS-T7RNAP with an empty plasmid (gray circles). Addition of NaF at 5 mM to the cultures is indicated with a vertical black arrow (F^-). Error bars correspond to standard deviations of three different biological replicates. **(B)** Intracellular concentration of 5'-FDA in the cells after 24 and 48 h of induction of the fluoride-dependent riboswitch with 5 mM NaF. CDW, cell dry weight. Data are presented as mean values and error bars correspond to standard deviations of three different biological replicates. Source data are provided as a Source Data file.

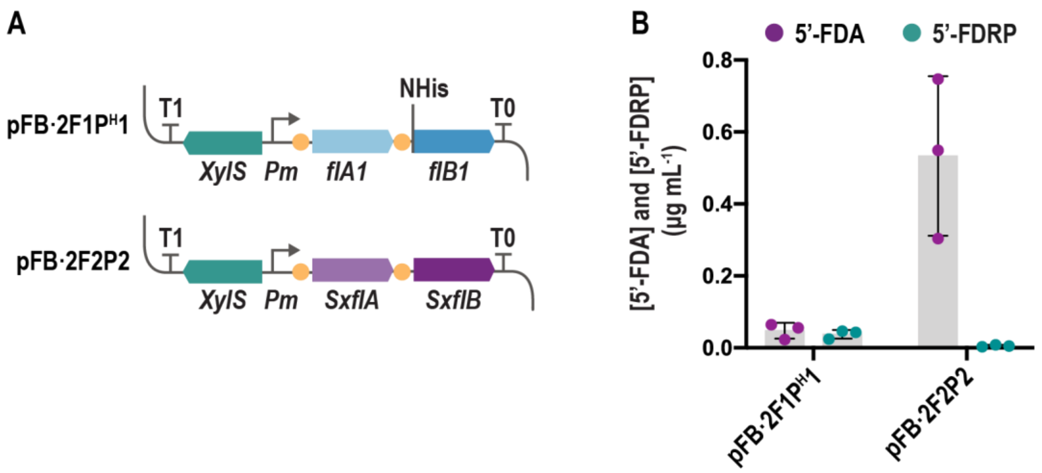

Supplementary Fig. 6. *In vitro* biosynthesis of 5'-fluoro-5'-deoxyadenosine and 5'-fluoro-5'-deoxy-D-ribose 1-phosphate. (A) Structure of plasmid constructs carrying the genes encoding fluorinases and phosphorylases from *Streptomyces* sp. MA37 (*flA1* and *flB1*) and *S. xinghaiensis* (*SxflA* and *SxflB*) under transcriptional regulation of the XylS/*Pm* expression system. FlB1 was tagged in the *N*-terminal domain using a 6× histidine tag (NHis); T0 and T1 indicate synthetic transcriptional terminators. **(B)** *In vitro* biosynthesis of 5'-fluoro-5'-deoxyadenosine (5'-FDA) and 5'-fluoro-5'-deoxy-D-ribose 1-phosphate (5'-FDRP) using cell-free extracts of *P. putida* KT2440 with selected fluorinases and phosphorylases in the configurations described in **(A)**. Data are presented as mean values and error bars correspond to the standard deviations of three different biological replicates. Source data underlying Supplementary Figure 6b are provided as a Source Data file.

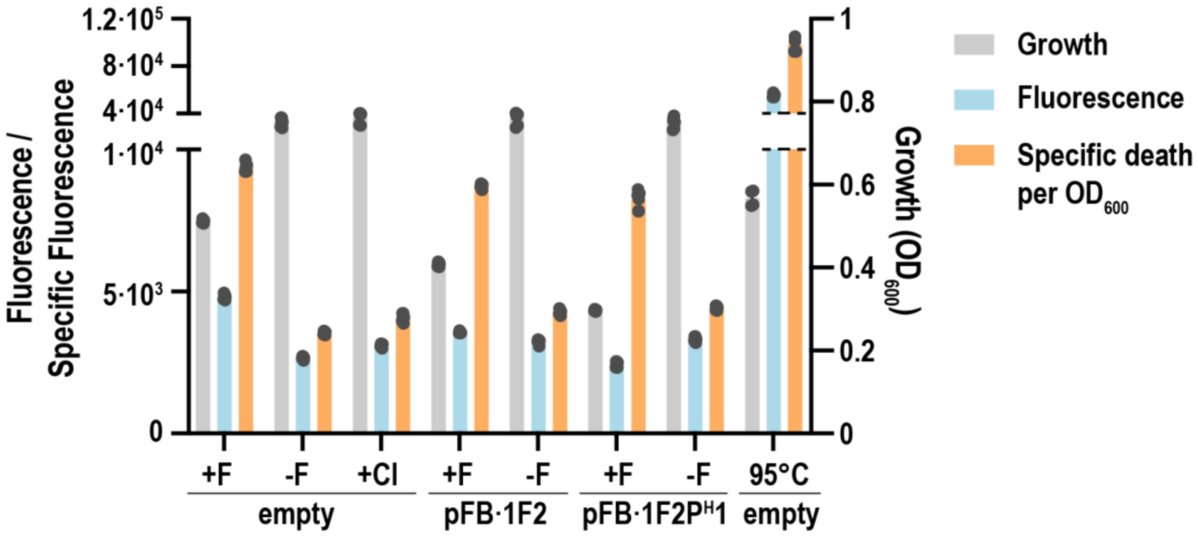

Supplementary Fig. 7. Quantification of alive and dead *P. putida* cells under biofluorination conditions. Cultures of *P. putida* KT2440::FRS-T7RNAP bearing plasmid pFB·1F2 (encoding a fluorinase from *S. xinghaensis*) or plasmid pFB·1F2P^{H}1 (encoding fluorinase and phosphorylase from *S. xinghaensis* and *Streptomyces* sp. MA37, respectively), were added or not with 15 mM NaF (indicated as +F and –F, respectively). Cells harvested from these cultures were treated with propidium iodide, and the fraction of dead cells was determined measuring fluorescence at an excitation/emission wavelength of 544/612 nm (blue bars). Growth was determined by measuring OD_{600} (grey bars), and the *specific death* was determined by normalizing the fluorescence reading corresponding to dead cells to the OD_{600} (orange bars). A culture of the same strain bearing an empty plasmid, added (+F) or not (–F) with NaF, was used as a control. Further controls included cultures supplemented with 15 mM NaCl (+Cl) or heated at 95°C for 5 min as indicated in the figure. Representative results from three different biological replicates are shown, and dots indicate individual measurements. Source data are provided as a Source Data file.

Supplementary References

1. Meselson, M. & Yuan, R. DNA restriction enzyme from *E. coli*. *Nature* **217**, 1110-1114 (1968).
2. Bagdasarian, M. *et al.* Specific purpose plasmid cloning vectors. II. Broad host range, high copy number, RSF1010-derived vectors, and a host-vector system for gene cloning in *Pseudomonas*. *Gene* **16**, 237-247 (1981).
3. Silva-Rocha, R. *et al.* The Standard European Vector Architecture (SEVA): a coherent platform for the analysis and deployment of complex prokaryotic phenotypes. *Nucleic Acids Res.* **41**, D666-D675 (2013).
4. Choi, K.H. *et al.* A Tn7-based broad-range bacterial cloning and expression system. *Nat. Methods* **2**, 443-448 (2005).
5. Calero, P., Jensen, S.I. & Nielsen, A.T. Broad-host-range *ProUSER* vectors enable fast characterization of inducible promoters and optimization of *p*-coumaric acid production in *Pseudomonas putida* KT2440. *ACS Synth. Biol.* **5**, 741-753 (2016).
6. Volke, D.C., Turlin, J., Mol, V. & Nikel, P.I. Physical decoupling of XylS/*Pm* regulatory elements and conditional proteolysis enable precise control of gene expression in *Pseudomonas putida*. *Microb. Biotechnol.* **13**, 222-232 (2020).
7. Aparicio, T., de Lorenzo, V. & Martínez-García, E. CRISPR/Cas9-based counterselection boosts recombineering efficiency in *Pseudomonas putida*. *Biotechnol. J.* **13**, e1700161 (2018).
8. Sánchez-Pascuala, A., de Lorenzo, V. & Nikel, P.I. Refactoring the Embden-Meyerhof-Parnas pathway as a whole of portable *GlucoBricks* for implantation of glycolytic modules in Gram-negative bacteria. *ACS Synth. Biol.* **6**, 793-805 (2017).
9. Ashton, T.D. & Scammells, P.J. An improved synthesis of 5'-fluoro-5'-deoxyadenosines. *Bioorg. Med. Chem. Lett.* **15**, 3361-3363 (2005).
