# Supplementary material for: A fluoride-responsive genetic circuit enables in vivo biofluorination in engineered Pseudomonas putida
Source: Nat Commun. 2020 Oct 7;11:5045. doi: 10.1038/s41467-020-18813-x (PMC7541441; doi:10.1038/s41467-020-18813-x)
Supplement: Supplementary file 3 — Reporting Summary [file 41467_2020_18813_MOESM3_ESM.pdf]

nature research

Corresponding author(s): Pablo I. Nikel

Last updated by author(s): Sep 5, 2020

Reporting Summary

Nature Research wishes to improve the reproducibility of the work that we publish. This form provides structure for consistency and transparency in reporting. For further information on Nature Research policies, see our Editorial Policies and the Editorial Policy Checklist.

Statistics

For all statistical analyses, confirm that the following items are present in the figure legend, table legend, main text, or Methods section.

n/a	Confirmed	
☐	☒	The exact sample size (*n*) for each experimental group/condition, given as a discrete number and unit of measurement
☐	☒	A statement on whether measurements were taken from distinct samples or whether the same sample was measured repeatedly
☐	☒	The statistical test(s) used AND whether they are one- or two-sided<br>*Only common tests should be described solely by name; describe more complex techniques in the Methods section.*
☒	☐	A description of all covariates tested
☒	☐	A description of any assumptions or corrections, such as tests of normality and adjustment for multiple comparisons
☐	☒	A full description of the statistical parameters including central tendency (e.g. means) or other basic estimates (e.g. regression coefficient) AND variation (e.g. standard deviation) or associated estimates of uncertainty (e.g. confidence intervals)
☐	☒	For null hypothesis testing, the test statistic (e.g. *F*, *t*, *r*) with confidence intervals, effect sizes, degrees of freedom and *P* value noted<br>*Give P values as exact values whenever suitable.*
☒	☐	For Bayesian analysis, information on the choice of priors and Markov chain Monte Carlo settings
☒	☐	For hierarchical and complex designs, identification of the appropriate level for tests and full reporting of outcomes
☒	☐	Estimates of effect sizes (e.g. Cohen's *d*, Pearson's *r*), indicating how they were calculated

Our web collection on statistics for biologists contains articles on many of the points above.

Software and code

Policy information about availability of computer code

Data collection	SynergyMX microtiter plate reader, ELx808 microtiter plate reader, NanoDrop ND-1000 UV-Vis Spectrophotometer, Cell homogenizer Precellys 24, French press Avestin Emulsiflex C5, SpeedVac concentrator, Prominence XR20 HPLC system, 5500 QTRAP mass spectrometer, 600 MHz Bruker AVANCE III HD spectrometer equipped with a Bruker BBFO SmartProbe, 800 MHz Bruker AVANCE III HD spectrometer equipped with a TCI Cryoprobe, Bruker Advance III HD 500 spectrometer equipped with a SmartProbe BBFO+ or TCI CryoProbe.
Data analysis	Data analysis was carried out with built-in software (LC-MS/MS and NMR, growth data), GraphPad Prism, MS Excel, ClustalW9, and PhyML 3.0

For manuscripts utilizing custom algorithms or software that are central to the research but not yet described in published literature, software must be made available to editors and reviewers. We strongly encourage code deposition in a community repository (e.g. GitHub). See the Nature Research guidelines for submitting code & software for further information.

Data

Policy information about availability of data

All manuscripts must include a data availability statement. This statement should provide the following information, where applicable:

- Accession codes, unique identifiers, or web links for publicly available datasets
- A list of figures that have associated raw data
- A description of any restrictions on data availability

Any supplementary information, chemical compound information and source data are available in the online version of the paper. Reprints and permissions information is available online at http://www.nature.com/reprints/index.html. Correspondence and requests for materials should be addressed to P.I.N.

Field-specific reporting

Please select the one below that is the best fit for your research. If you are not sure, read the appropriate sections before making your selection.

- [x] Life sciences
- [ ] Behavioural & social sciences
- [ ] Ecological, evolutionary & environmental sciences

For a reference copy of the document with all sections, see nature.com/documents/nr-reporting-summary-flat.pdf

Life sciences study design

All studies must disclose on these points even when the disclosure is negative.

Sample size	Each experiment was carried out in replicates as indicated below. No statistical methods were used to predefine sample size. For each type of experiment, sample size was chosen based on the common methods in the literature and based in our preliminary experiments. All experiments were carried out in biological replicates, which is the preferred approach in the field.
Data exclusions	No data were excluded from the analysis.
Replication	All experiments were done at least in three independent biological replicates with at least two technical replicates each. All attempts of replication were successful.
Randomization	All relevant samples were compared to their corresponding controls, so no randomization was necessary. All experiments were carried out with bacterial colonies randomly picked from agar plates, which prevents any bias.
Blinding	Blinding is not required for this study. Group allocation is not part of our experimental design. blinding during group allocation is irrelevant because the samples of bacterial cultures that were split into different conditions were random samplings and there is no control over which cells will be selected. Thus, there is no bias during group allocation.

Reporting for specific materials, systems and methods

We require information from authors about some types of materials, experimental systems and methods used in many studies. Here, indicate whether each material, system or method listed is relevant to your study. If you are not sure if a list item applies to your research, read the appropriate section before selecting a response.

Materials & experimental systems

n/a	Involved in the study	
☒	☐	Antibodies
☒	☐	Eukaryotic cell lines
☒	☐	Palaeontology and archaeology
☒	☐	Animals and other organisms
☒	☐	Human research participants
☒	☐	Clinical data
☒	☐	Dual use research of concern

Methods

n/a	Involved in the study	
☒	☐	ChIP-seq
☒	☐	Flow cytometry
☒	☐	MRI-based neuroimaging
