# Supplementary material for: A fluoride-responsive genetic circuit enables in vivo biofluorination in engineered Pseudomonas putida
Source: Nat Commun. 2020 Oct 7;11:5045. doi: 10.1038/s41467-020-18813-x (PMC7541441; doi:10.1038/s41467-020-18813-x)
Supplement: Supplementary file 4 — Description of Additional Supplementary Files [file 41467_2020_18813_MOESM4_ESM.pdf]

Description of Additional Supplementary Files

File name: Supplementary Data 1

Description: DNA sequences of the fluoride-dependent riboswitches used in this study
