# Supplementary material for: A fluoride-responsive genetic circuit enables in vivo biofluorination in engineered Pseudomonas putida
Source: Nat Commun. 2020 Oct 7;11:5045. doi: 10.1038/s41467-020-18813-x (PMC7541441; doi:10.1038/s41467-020-18813-x)
Supplement: Supplementary file 2 — Peer Review File [file 41467_2020_18813_MOESM2_ESM.pdf]

Editorial Note: This manuscript has been previously reviewed at another journal that is not operating a transparent peer review scheme. This document only contains reviewer comments and rebuttal letters for versions considered at *Nature Communications.*

REVIEWERS' COMMENTS:

Reviewer #1 (Remarks to the Author):

Thank you for addressing my comments and improving the accuracy and clarity of your claims. The manuscript is well-written and neatly highlights the engineering of a recombinant host to produce fluorometabolites with low toxicity.

Minor comments:
Pg 5, line 16: This sentence can safely end at “3.8-fold induction”. The subsequent clause does not add extra information and may be confusing.

The SM title needs to be updated to reflect the new title

Reviewer #2 (Remarks to the Author):

The authors provided a mass spectrometric analysis of 5′-fluoro-5′-deoxyadenosine and 5′-fluoro-5′-deoxy-D-ribose-1-phosphate in Supporting Information, which now also contains sufficient information for the detection and quantification of fluorinated nucleotides and sugars. In addition, these results are also discussed in a revised manuscript.

Reviewer #3 (Remarks to the Author):

The authors have addressed the issues I raised in my previous review. The only other comment to make is the following:

The sentence "As such, the development of a biotechnology for the site-selective introduction of F into structurally-diverse molecules..." (specifically the "a biotechnology") is awkward and should be rephrased in a more eloquent way.

[Editor: Reviewer #4 is unavailable. We asked Reviewer #3 to comment your response. Reviewer #3 thinks that most issues have been addressed. However, (s)he asks to discuss the influence of promoters in response to Reviewer #3 second comment. This request was stated in Remark to Editor section.]

Response to the referees' comments and how they have been addressed in the revised version (in blue)

Reviewer # 1
Thank you for addressing my comments and improving the accuracy and clarity of your claims. The manuscript is well-written and neatly highlights the engineering of a recombinant host to produce fluorometabolites with low toxicity.
Thanks for the positive evaluation of our contribution.

Page 5, line 16: This sentence can safely end at "3.8-fold induction". The subsequent clause does not add extra information and may be confusing.
Done.

The SM title needs to be updated to reflect the new title.
As per the editor's request, we have kept the original title for our manuscript.

Reviewer # 2
The authors provided a mass spectrometric analysis of 5'-fluoro-5'-deoxyadenosine and 5'-fluoro-5'-deoxy-D-ribose-1-phosphate in Supporting Information, which now also contains sufficient information for the detection and quantification of fluorinated nucleotides and sugars. In addition, these results are also discussed in a revised manuscript.
Thanks for the endorsement of our article.

Reviewer # 3
The authors have addressed the issues I raised in my previous review.
Thanks for your support.

The only other comment to make is the following: the sentence "As such, the development of a biotechnology for the site-selective introduction of F into structurally-diverse molecules..." (specifically the "a biotechnology") is awkward and should be rephrased in a more eloquent way.
Modified as 'The development of approaches for the site-selective introduction of F into structurally-diverse molecules would circumvent the harsh chemical methods associated with its introduction in organic chemistry, and remains one of the great goals in metabolic engineering' in Page 3, lines 11-14.

Editor: Reviewer # 4 is unavailable. We asked Reviewer # 3 to comment your response. Reviewer # 3 thinks that most issues have been addressed. However, (s)he asks to discuss the influence of promoters in response to Reviewer # 3's second comment. This request was stated in Remark to Editor section. Thanks for the comment. To facilitate the discussion, we copy the original comment by Reviewer # 3 and our response below:

[2] For *in vitro* biofluorination in engineered *P. putida* as shown in Fig. 2E and 2F, genes encoding fluorinases from *Streptomyces* sp. MA37 (*flA1*) and *S. xinghaiensis* (*SxflA*) were combined with XylS/*Pm* expression system or the FRS-T7RNA polymerase circuit. 5'-FDA production in pFB.2F2 was higher than in pFB.1F2, which illustrated that the XylS/*Pm* system is more effective. However, 5'-FDA production with pFB.2F1 with XylS/*Pm* is similar to or even lower than pFB.1F1. What is the reason for such results? Please keep the same order for plasmids shown in Fig. 2E and 2F.
We apologize that we have unintentionally misled the Reviewer. The order of constructs in Figs. 2E and F has been corrected as suggested. Differences in 5'-FDA content across conditions have

been statistically compared (and all experimental data added to Fig. 2F, including new replicates done for this purpose) to illustrate the fact that *SxflA* outperforms *flA1* under control of both XylS/*Pm* and the FRS-T7 RNA polymerase circuit. We also note that 3-methylbenzoate (the inducer of the XylS/*Pm* system) was observed to exert some toxicity on the engineered cells, which was another reason to keep the synthetic FRS-T7 RNA polymerase circuit for further experiments. The text has been edited to highlight the purpose and conclusion of this experiment as suggested (P8).

Additional discussion for the current version of our article: As we has indicated previously, this apparent behavior is probably due to the relatively large variability of 5'-FDA output when using either expression system, and the difference in 5'-FDA titers between experiments with XylS/*Pm*- and FRS-T7RNAP/P_{T7} circuit-based expression of the fluorinase gene is not statistically significant (see Page 8, lines 18-20). This does not detract any of our claims: we kept the FRS-T7RNAP/P_{T7} circuit as it enabled the highest fluorometabolite titers across all experiments tested in our article, and also because we have observed some toxicity issues when using 3-methylbenzoate as inducer of the XylS/*Pm* system (Page 8, lines 24-26). We have modified the article in these places to indicate all these observations.
